# AHR/NRF2 Dual Agonist Prediction and Natural Compound Screening Based on Machine Learning: A New Strategy for the Treatment of Atopic Dermatitis

**DOI:** 10.3390/ijms27083530

**Published:** 2026-04-15

**Authors:** Yu Zhen, Qi Li, Xiaoxu Hu, Xiaorui Liu, Zhijie Shao, Heidi Qunhui Xie, Bin Zhao, Li Xu

**Affiliations:** 1 State Key Laboratory of Environmental Chemistry and Ecotoxicology, Research Center for Eco-Environmental Sciences, Chinese Academy of Sciences, Beijing 100085, China; zhenyu22@mails.ucas.ac.cn (Y.Z.); liqi17liqi@163.com (Q.L.); gracehu97@foxmail.com (X.H.); liuxiaorui24@mails.ucas.ac.cn (X.L.); perseids7@outlook.com (Z.S.); qhxie@rcees.ac.cn (H.Q.X.); 2 Sino-Danish College & Sino-Danish Centre for Education and Research, University of Chinese Academy of Sciences, Beijing 100190, China; 3 Department of Pharmacy, University of Copenhagen, 2100 Copenhagen, Denmark; 4 School of Environment, Hangzhou Institute for Advanced Study, University of Chinese Academy of Sciences, Hangzhou 310024, China; binzhao@ucas.ac.cn; 5 University of Chinese Academy of Sciences, Beijing 100049, China

**Keywords:** AHR, NRF2, atopic dermatitis, machine learning, natural compounds

## Abstract

In the treatment of atopic dermatitis (AD), synergistic activation of the aryl hydrocarbon receptor (AHR)/nuclear factor erythroid 2-related factor 2 (NRF2) pathways represents a promising strategy. However, known dual agonists are limited, and traditional screening methods are inefficient. Therefore, this study developed machine learning models to predict AHR/NRF2 dual agonists using molecular descriptors and fingerprints. All models achieved area under the receiver operating characteristic curve (*AUC*) values above 0.86, indicating good classification performance. The optimal AHR model showed an accuracy (*ACC*) of 0.811 and an *AUC* of 0.878, while the best NRF2 model yielded an *ACC* of 0.839 and an *AUC* of 0.907. Based on this model, compounds with a low fraction of sp^3^-hybridized carbons, moderate hydrophobicity, limited alkyl chains, and highly conjugated structures tend to act as AHR/NRF2 dual agonists. Finally, this study screened 1011 potential natural AHR/NRF2 dual agonists suitable for drug development. Among these, 2-arylbenzofurans, alkaloids, phenanthrenes, flavones, and furocoumarins demonstrated particular advantages. For validation, Indirubin, imperatorin and 3′-O-Methylbutastatin III were first discovered as AHR/NRF2 dual agonists in HaCaT cells. This work provides a robust predictive tool, clarifies key molecular features of dual agonists, and may support the discovery of anti-AD agents.

## 1. Introduction

The aryl hydrocarbon receptor (AHR) is an evolutionarily ancient ligand-activated transcription factor highly expressed in all skin cell types [1]. It regulates numerous genes critical for fundamental skin functions, including environmental toxin metabolism [2], keratinocyte differentiation, epidermal barrier function [3,4], melanogenesis [5], and skin immunity and inflammatory responses [6,7]. Consequently, a certain level of AHR activity is essential for maintaining skin integrity and adapting to acute stress conditions. The nuclear factor erythroid 2-related factor 2 (NRF2) signaling pathway is a crucial intracellular antioxidant and stress response pathway, with downstream antioxidant enzymes including NAD(P)H quinone dehydrogenase 1 (NQO1), heme oxygenase-1 (HO-1), glutathione s-transferase (GST), catalase (CAT), superoxide dismutase (SOD), and others [8]. NRF2 activation protects skin cells from H_2_O_2_-induced and UV-induced cellular damage, including oxidative stress [9], DNA damage [10], apoptotic injury [11], mitochondrial dysfunction [12], inflammation [12], skin aging [13,14], and even skin cancer [15]. Crosstalk between AHR and NRF2 remains understudied. Although evidence suggests that NRF2 acts as a downstream target of AHR [16] or that AHR may indirectly activate NRF2 via reactive oxygen species generated by cytochrome P450 1A1 (CYP1A1) [17], this regulatory process appears to be species- and cell-dependent.

Given the critical roles of AHR and NRF2 in the skin, recent studies suggest these pathways may serve as therapeutic targets for atopic dermatitis (AD). AD is characterized by a Th2-polarized immune response with elevated levels of interleukin-4 (IL-4) and interleukin-13 (IL-13) [6]. Dysfunction of the skin barrier is highly correlated with AD pathogenesis, and a key downstream target of AHR is the epidermal differentiation complex [18]. For example, topical application of coal tar or glyteer can restore expression of the skin barrier protein filaggrin (FLG) in AD patients’ skin by activating AHR [3,19,20]. Both AHR and NRF2 activation can attenuate Th2 inflammation [21] while NRF2 simultaneously initiates antioxidant pathways to reduce oxidative stress and inhibit signal transducer and activator of transcription 6 (STAT6) activation [20]. Therefore, dual agonists targeting both AHR and NRF2 hold significant potential as novel therapeutic targets for AD. Currently known dual agonists such as tapinarof, coal tar, and WBI-1001 have demonstrated efficacy in treating AD [18]. Furthermore, current treatments such as glucocorticoids and monoclonal antibodies are often limited by management difficulties and adverse side effects, which make natural compound-derived drugs increasingly attractive due to their greater bioactivity and safety.

However, given the vast diversity of natural compounds, identifying dual agonists of AHR/NRF2 through experimental approaches is time-consuming, labor-intensive, and costly. Fortunately, with advances in computer science, machine learning-supported quantitative structure-activity relationship (QSAR) methods have demonstrated tremendous promise in small-molecule drug discovery [22]; these approaches correlate molecular descriptors of chemical structures with their biological activities or responses [23]. Among various molecular descriptors, molecular fingerprints can effectively and simply represent molecular structures, physicochemical properties, and pharmacophore characteristics in a 2D format [24]. They are widely applied in diverse drug discovery processes, including virtual screening, similarity-based compound searches, and drug ADMET (absorption, distribution, metabolism, excretion, and toxicity) prediction [25]. Currently, multiple algorithms from ensemble learning and deep learning have been applied to predict agonists for various targets, such as gamma-aminobutyric acid type A receptor (GABAA) [23], estrogen receptors [26], G protein-coupled receptors (GPCRs) [27], and peroxisome proliferator-activated receptor delta (PPAR-δ) [28]. However, existing machine learning studies on AHR agonists have yet to reveal structure-activity relationships between molecular structures and biological activities. Furthermore, no relevant literature has been reported in the field of machine learning research for NRF2 agonists.

This study primarily established machine learning models for predicting AHR/NRF2 dual agonists, compared the performance of different models, explored structural and chemical features of dual agonists through feature importance analysis, and screened several unreported natural compounds. This study successfully developed a reliable prediction tool with high accuracy, significantly reducing experimental costs. It not only elucidated the molecular properties of AHR/NRF2 dual agonists but also provided a convenient and robust framework for screening drugs targeting atopic dermatitis.

## 2. Results

### 2.1. Statistical Analysis of RDKit Descriptors

**Figure 1:**
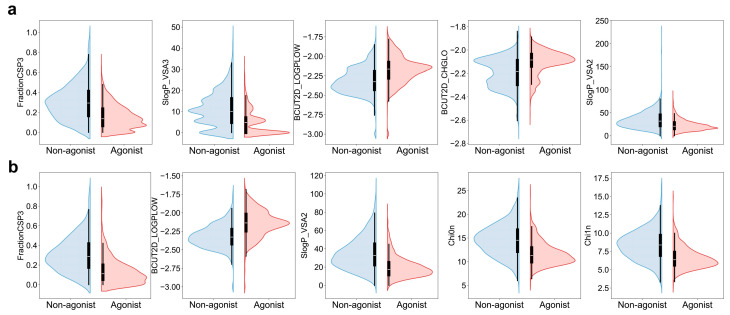
Distribution of the top five RDKit descriptors with the highest point-biserial correlation to AHR and NRF2 agonist activity. (**a**) Violin plots showing the distribution of the top five descriptors (FractionCSP3, SlogP_VSA3, BCUT2D_LOGPLOW, BCUT2D_CHGLO, SlogP_VSA2) for AHR agonists (red) and non-agonists (blue); (**b**) Violin plots showing the distribution of the top five descriptors (FractionCSP3, BCUT2D_LOGPLOW, SlogP_VSA2, Chi0n, Chi1n) for NRF2 agonists (red) and non-agonists (blue). The black box inside each violin represents the interquartile range, with the vertical line indicating the median value. The shape of the violin reflects the kernel density estimate of the data distribution, highlighting differences in central tendency and spread between agonist and non-agonist groups.

After preprocessing, the AHR dataset contained 5192 agonists and 5192 non-agonists, while the NRF2 dataset contained 1242 agonists and 1242 non-agonists. Molecular descriptors for each compound were obtained using RDKit, and point-biserial correlation coefficients were calculated to explore differences between agonists and non-agonists. Figure 1 lists the top five descriptors ranked by point-biserial correlation coefficient. Specifically, for AHR, agonists are characterized by higher values of BCUT2D-CHGLO and BCUT2D-LOGPLOW, whereas non-agonists exhibit higher values of FractionCSP3, SlogP_VSA2, and SlogP_VSA3. For NRF2, agonists are associated with higher BCUT2D-LOGPLOW, while non-agonists tend to show higher values of FractionCSP3, SlogP_VSA2, Chi0n, and Chi1n. FractionCSP3 denotes the proportion of sp^3^-hybridized carbon atoms in the molecule relative to the total number of carbon atoms. BCUT2D-LOGPLOW characterizes molecular hydrophilicity, while SlogP_VSA2 and SlogP_VSA3 both characterize hydrophobicity within the molecule [29,30]. BCUT2D-CHGLO represents the charge distribution of the molecule, while Chi0n and Chi1n denote the average values of the net charge. Thus, for AHR and NRF2, molecules with a lower proportion of sp^3^ hybridization and moderate hydrophobicity are more likely to act as agonists. Additionally, AHR agonists are characterized by stronger electrophilicity.

### 2.2. Prediction Performance of the Classification Models

**Figure 2:**
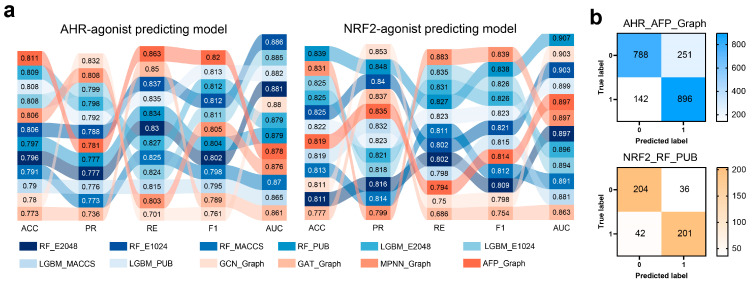
Evaluation of classification performance of models for AHR and NRF2 agonist prediction. (**a**) Performance metrics (Accuracy (*ACC*), Precision (*PR*), Recall (*RE*), F_1_-score (*F*_1_), and Area Under the ROC Curve (*AUC*)) of multiple machine learning models. Numerical values are labeled to compare model performance across metrics. (**b**) Confusion matrices for the top-performing models (AHR_AFP_Graph and NRF2_RF_PUB), showing the counts of true negatives, false positives, false negatives, and true positives. Color intensity reflects sample size. Models include random forest (RF), light gradient boosting machine (LGBM), and graph neural networks (GCN, GAT, MPNN, AFP) using different molecular fingerprints (E2048, E1024, MACCS, PUB) or graph representations.

Molecular fingerprints, including extended-connectivity fingerprint with a diameter of 4 (ECFP4)-2048 bits (E2048), ECFP4-1024 bits (E1024), PubChem (PUB), and Molecular access system keys (MACCS), were used to train Random Forest (RF) and Light Gradient Boosting Machine (LGBM) classifiers. Graph convolutional network representations of molecules (abbreviated as Graph) were employed to train four neural network models: Graph Convolutional Networks (GCNs), Graph Attention Networks (GATs), Message Passing Neural Networks (MPNN), and AttentiveFP (AFP). A total of 24 models were constructed and compared for both AHR and NRF2. Results from fivefold cross-validation on the training set are presented in the Supplementary Materials (Supplementary Table S4). Figure 2a lists the precision (*PR*), recall (*RE*), accuracy (*ACC*), and *F*_1_, which are typical indicators, and the area under the receiver operating characteristic curve (*AUC*) metrics for the 24 models on the test set. Notably, all models achieved *AUC* values above 0.86, indicating strong classification performance. Among the neural network models, GAT and GCN performed the worst. Across all four molecular fingerprints, MACCS consistently yielded the lowest scores, suggesting that the MACCS fingerprint may not adequately represent molecular structures. Assuming *ACC* as the evaluator, the best-performing classification model for AHR agonists is AHR_AFP_Graph (*ACC* = 0.811, *AUC* = 0.878); for NRF2 agonists, the top model is NRF2_RF_PUB (*ACC* = 0.839, *AUC* = 0.907) (the naming rules for the model can be found in Section 4). Figure 2b displays the confusion matrices of these two top models on the test set. It shows balanced sample sizes for labels 0 and 1, with consistent performance across agonists and non-agonists, demonstrating the models’ excellent classification capability.

### 2.3. Shapley Additive Explanations of Molecular Fingerprints

**Figure 3:**
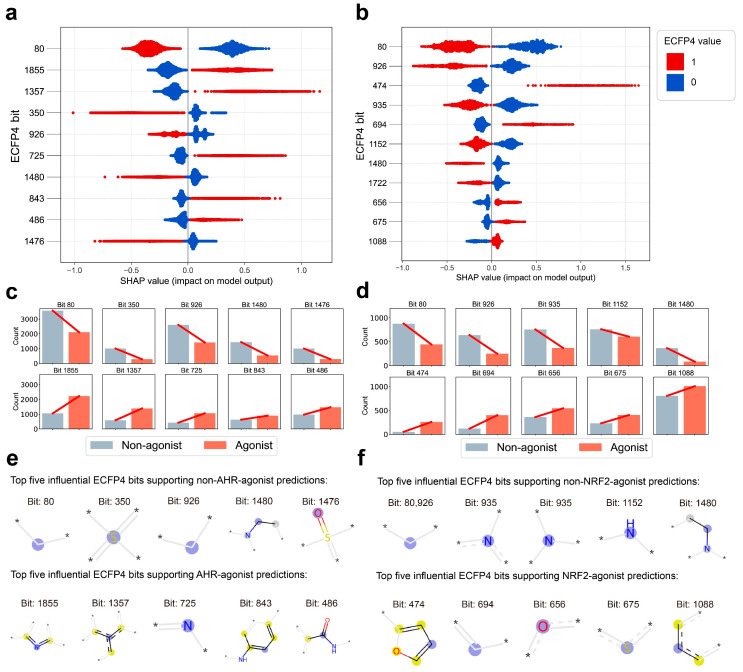
SHAP analysis and visualization of key ECFP4 fingerprint bits driving AHR and NRF2 agonist predictions. (**a**) SHAP summary plot for the AHR_LGBM_E2048 model, showing the impact of the top 10 most influential ECFP4 fingerprint bits on the model output. The x-axis represents SHAP values (positive values promote agonist prediction, negative values promote non-agonist prediction), while the y-axis lists ECFP4 bit indices. Color coding indicates the ECFP4 bit value (red = 1, bit present; blue = 0, bit absent), revealing how each fingerprint feature influences model decisions. (**b**) SHAP summary plot for the NRF2_LGBM_E2048 model, formatted identically to panel (**a**), highlighting the top 10 ECFP4 bits driving NRF2 agonist/non-agonist predictions. (**c**) Bar chart of ECFP4 bit occurrence frequencies in AHR agonists (orange) and non-agonists (grey) for the top 10 influential bits identified in panel (**a**). (**d**) Bar chart of ECFP4 bit occurrence frequencies in NRF2 agonists (orange) and non-agonists (grey) for the top 10 influential bits identified in panel (**b**). (**e**) 2D molecular visualization of the top five ECFP4 bits supporting non-AHR-agonist predictions (top row) and AHR-agonist predictions (bottom row) in the AHR_LGBM_E2048 model. Atoms are colored by type: blue = central atom, yellow = aromatic atoms, gray = aliphatic atoms. Asterisks (*) denote omitted portions of the molecular substructure. (**f**) 2D molecular visualization of the top five ECFP4 bits supporting non-NRF2-agonist predictions (top row) and NRF2-agonist predictions (bottom row) in the NRF2_LGBM_E2048 model, using the same atom coloring scheme as panel (**e**) to highlight relevant chemical substructures.

To investigate which molecular structures play key roles in distinguishing agonists from non-agonists, the SHAP explainable machine learning library was employed for analysis. Since E2048 provides more comprehensive and fine-grained molecular information, and the LGBM models based on these fingerprints demonstrated superior performance (AHR_LGBM_E2048: *ACC* = 0.809, *AUC* = 0.879; NRF2_LGBM_E2048: *ACC* = 0.825, *AUC* = 0.896), AHR_LGBM_E2048 and NRF2_LGBM_E2048 were selected for subsequent analysis. Figure 3a,b illustrate molecular fingerprint bits contributing most significantly to the model. Each point represents a sample, with color indicating whether the structure corresponding to the fingerprint bit is present. The SHAP value on the x-axis indicates whether the fingerprint bit exerts a positive or negative influence on the prediction for each sample. A larger absolute value of the SHAP value signifies a greater contribution to the model. For both models, only one color appears on either side of the SHAP value of “0”, indicating that the direction of influence of these features is very clear. This further demonstrates the models’ excellent ability to classify agonists and non-agonists based on these features.

For the AHR model, samples with a “1” at bits 80, 350, 926, 1476, and 1480 tend to be predicted as non-agonists, and the SHAP values of these features are relatively concentrated, indicating a stable effect. In contrast, samples with a “1” at bits 1855, 1357, 725, 843, and 486 are more likely to be predicted as agonists, and the SHAP values of these features are more dispersed, suggesting strong interactions between features—that is, the effect of these substructures depends on the presence of other substructures. For the NRF2 model, samples with a “1” at bits 80, 926, 935, 1152, 1480, and 1722 tend to be predicted as non-agonists, whereas samples with a “1” at bits 474, 694, 656, 675, and 1088 tend to be predicted as agonists. The patterns of concentration and dispersion of SHAP values are similar to those observed in the AHR model. Across the entire dataset, substructures that clearly support non-agonist predictions occur less frequently in agonists than in non-agonists, whereas substructures that favor agonist predictions are markedly more frequent in agonists (Figure 3c,d). However, no single substructure shows an absolute predominance, indicating that predicting agonists requires consideration of a combination of various substructures as well as the complex binding sites of large molecules.

To further clarify the specific structures corresponding to these important fingerprint bits, the top five features with positive and negative contributions were visualized using the RDKit library (Figure 3e,f). Considering that the same ECFP4 fingerprint position may correspond to different substructures in different molecules, we randomly selected 40 samples in which the bit value was “1”. The results indicate that despite variations in individual samples, the vast majority of samples exhibit identical or highly similar structures at the same fingerprint bit. Therefore, the structure with the highest frequency of occurrence was selected as the representative structure for display. For AHR, highly conjugated aromatic structures such as aromatic amides, aromatic amines, imines, and nitrogen-containing heterocycles (pyrrole, pyridine) are more likely to act as agonists. For NRF2, highly conjugated aromatic structures like furan and aromatic heterocycles are more likely to act as agonists. In contrast, hydrophobic substructures formed by nonpolar alkyl side chains are unfavorable for activating AHR and NRF2, consistent with the results presented in Section 2.1.

### 2.4. Potential Dual Agonists of AHR and NRF2 from the Natural Compound Library

**Figure 4:**
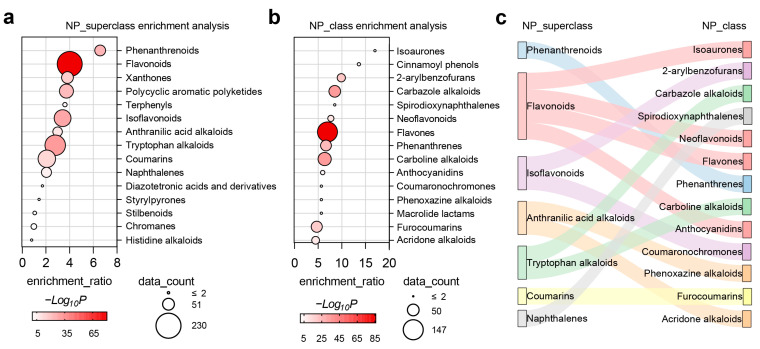
Enrichment analysis of superclass (**a**) and class (**b**) for potential AHR/NRF2 dual-agonist natural compounds, along with their hierarchical correspondence (**c**). The enrichment ratio represents the proportion of compounds in this category within the 1011 compounds relative to that in the entire dataset (NP: natural products).

Virtual screening of the COCONUT natural compound library was performed using the two best-performing models to identify dual agonists for AHR and NRF2. Following multiple rounds of screening of 695,142 compounds, 1011 potential natural small-molecule dual agonists with drug development potential were ultimately identified. A literature search via PubMed revealed that among these 1011 compounds, 79 have been reported to activate AHR, 129 to activate NRF2, and 43 to activate both AHR and NRF2. Detailed information on all 1011 compounds is provided in the supplementary table for reference (see Dataset S1: Predicted Dual AHR/NRF2 Agonists). These compounds represent highly promising AHR/NRF2 dual agonists, warranting further experimental validation. Additionally, enrichment analysis was performed on the categories of these 1011 potential natural dual agonists compared to the entire dataset. Results indicate that natural products such as phenanthrenoids, flavonoids, xanthones, polycyclic aromatic polyketides, isoflavonoids, alkaloids, and coumarins show greater potential as dual agonists of AHR/NRF2 (Figure 4a,b). At a more granular level, Figure 4c illustrates the corresponding relationships between categories. Subgroups such as 2-arylbenzofurans among isoflavonoids, carbazole alkaloids and carboline alkaloids among alkaloids, phenanthrenes among phenanthrenoids, flavones among flavonoids, and furocoumarins among coumarins exhibit greater advantages as dual agonists for AHR/NRF2. The molecular structural features of 2-arylbenzofurans, coumarins, alkaloids, and flavones further validate the results in Section 2.3.

### 2.5. Experimental Validation of Dual Agonists in HaCaT Cells

**Figure 5:**
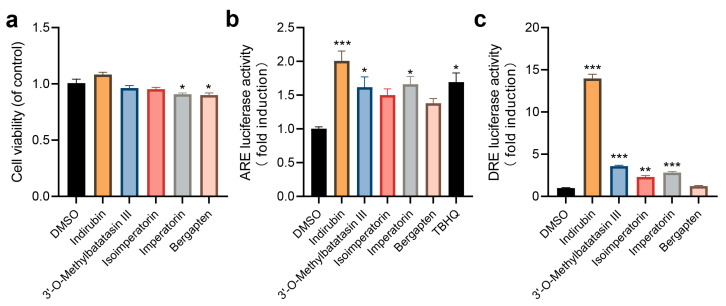
Validation of five natural compounds in HaCaT cells. (**a**) Cell viability test; (**b**) Detection of NRF2 pathway activation using dual luciferase reporter gene assay, with TBHQ used as a positive control; (**c**) Detection of AHR pathway activation using dual luciferase reporter gene assay, with indirubin serving as a positive control. The concentration of all compounds is 10 μM. Data are represented as mean ± SEM. Statistical significance is shown as * *p* < 0.05, ** *p* < 0.01, and *** for *p* < 0.001, as evaluated by one-way ANOVA.

Considering the availability of compounds and their anti-inflammatory potential in the con-text of traditional Chinese medicine, five natural compounds that have not been reported as AHR or NRF2 agonists were selected. Among them, isoimperatorin, imperatorin, and 3′-O-Methylbutastatin III have not been reported to activate AHR, while Indirubin, bergapten, and 3′-O-Methylbutastatin III have not been reported to activate NRF2. Due to the fact that human keratinocytes are commonly used in dermatitis research, five compounds were validated on the HaCaT cell line. First, the effects of 10 μM of various compounds on cell viability were tested. Cell viability remained above 90% in all groups, suggesting that these compounds did not exert cytotoxic effects in HaCaT cells (Figure 5a). Then, regarding the activation of the pathways, indirubin, imperatorin and 3′-O-Methylbutastatin III significantly activated AHR and NRF2, which are reported here for the first time as dual agonists. Isoimperatorin was found for the first time to significantly activate AHR. Although bergapten was not significant, it still showed a numerical trend of activating AHR and NRF2, and is a potential dual agonist. In summary, these results demonstrate that the machine learning model established in this study has great potential for screening dual agonists.

## 3. Discussion

In this study, machine learning prediction models for AHR/NRF2 agonists were established, achieving classification performance with AUC values above 0.86. While some studies have reported on machine learning-based screening for AHR agonists, these studies exhibit several limitations. Zhu et al. [31] employed neural networks and RF to construct predictive models (sample size = 8164). However, due to class imbalance in the training data, the models exhibited significantly different prediction accuracies for agonists and non-agonists. Meanwhile, Wojtyło et al. [32] optimized classification algorithms (sample size = 978), but achieved a maximum accuracy of 0.76. Furthermore, neither study thoroughly investigated the influence of molecular structural features. To address these issues, this study employed a dataset with a sufficient sample size and balanced categories (sample size = 10,384, agonist:non-agonist = 1:1). The constructed classification model achieved a maximum *ACC* of 0.811, significantly outperforming previous studies. Furthermore, by integrating molecular descriptor analysis, this study enhanced model interpretability and achieved the first machine learning-based screening of NRF2 agonists (NRF2_RF_PUB: *ACC* = 0.839), providing a more reliable predictive tool for computer-aided drug design targeting this pathway.

This study investigated the molecular structural characteristics of AHR/NRF2 agonists from three perspectives: statistical differences in RDKit descriptors, ECFP4 feature importance analysis, and natural compound screening. Results indicate that dual agonists exhibit significant structural commonalities, including a low fraction of sp^3^-hybridized carbons, moderate hydrophobicity, limited alkyl chains, and highly conjugated structures. Collectively, the presence of saturated carbons or localized alkyl chains within aromatic compounds appears to hinder activation of both pathways. This conclusion is consistent with previous studies suggesting that AHR and NRF2 ligands require planar aromatic rings and extended π-conjugated systems—where planarity essentially reflects the high proportion of sp^2^-hybridized carbon atoms in the benzene ring side chains and the integrity of the conjugated structure [33,34]. Furthermore, regarding AHR agonists, SHAP analysis revealed the critical role of nitrogen-containing functional groups in their structure: these groups form hydrogen bonds to establish specific binding with the receptor [35,36]. Based on these findings, this study is the first to explicitly propose specific compound categories—aromatic amides, aromatic amines, imines, and nitrogen-containing heterocycles (e.g., pyrrole, pyridine)—as structural guidelines for AHR ligand design, thereby complementing existing theoretical frameworks.

Additionally, given the critical role of the AHR-NRF2 axis in AD treatment, a panel of natural compounds was screened for dual agonists using established models. Currently, aside from the marketed drug tapinarof, few therapeutic agents targeting the AHR/NRF2 pathway for AD have been reported. Coal tar, one of the oldest therapies, restores expression of key skin barrier proteins via AHR and activates NRF2 by dephosphorylating STAT6 to disrupt Th2 cytokine signaling. However, questions remain regarding its safety and potential carcinogenicity [20]. Difamilast treatment inhibits IL-33 activity via the AHR/NRF2 pathway, contributing to improved AD symptoms [37]. Ketoconazole (KCZ) suppresses IL-8 production and exhibits cell-protective effects mediated by the AHR/NRF2 system [38]. The only natural compound reported is triacylglycerol from the cannabis plant, which alleviates nicotinamide adenine dinucleotide phosphate oxidase 2 (NOX2)-dependent mitochondrial dysfunction and repairs the skin barrier via the AHR/NRF2 pathway, making it a promising therapeutic agent for preventing and treating AD [39]. Tapinarof, as a natural compound, demonstrates clear therapeutic efficacy, fully confirming that natural compounds targeting the AHR/NRF2 pathway possess potential therapeutic value for AD while exhibiting favorable safety profiles. This study has identified some new AHR/NRF2 dual agonists, providing more possibilities for the treatment of AD. However, this is only a small part of the screening results, and further validation and exploration of their potential for the treatment of AD are needed in the future. Although numerous research gaps remain in this field, this study proposed 2-arylbenzofurans, carbazole alkaloids, carboline alkaloids, phenanthrenes, flavones, and furocoumarins as potential dual agonists, providing crucial directional guidance and theoretical reference for subsequent research in related fields.

The rapid advancement of artificial intelligence (AI) in recent years has enabled algorithms to shine in the field of life sciences, with virtual drug screening emerging as a prevailing trend [40]. Machine learning has significantly propelled drug discovery [41]. However, the models established in this study still hold room for improvement. Targets often possess multiple binding sites that drive distinct downstream responses. For instance, AHR is commonly recognized as the receptor for dioxins and polycyclic aromatic hydrocarbons (PAHs), leading to its exclusion or abandonment in drug development [42,43]. Nevertheless, certain endogenous ligands of AHR are essential for maintaining normal physiological functions, and activation of AHR by some natural compounds can yield beneficial effects [44]. Therefore, in agonist screening, machine learning should distinguish compounds that produce different downstream effects. This, however, requires further elucidation of the AHR protein structure and its co-crystal complexes with ligands. Beyond machine learning, more intelligent algorithms are emerging with AI advancements, such as self-learning multimodal large models for DNA, RNA, and protein tasks [45]. The screening of agonists or drug candidates can evolve toward more complex and intelligent approaches in the future. This will not only better replace labor-intensive and resource-consuming experiments but also provide insights into the underlying biological mechanisms.

## 4. Materials and Methods

### 4.1. Dataset

The datasets for AHR and NRF2 agonists are both from PubChem (https://pubchem.ncbi.nlm.nih.gov/bioassay/2796 (accessed on 25 December 2023) and https://pubchem.ncbi.nlm.nih.gov/bioassay/624171 (accessed on 6 January 2025)), which include the molecular formula and test results of each compound. The data were preprocessed by removing inorganic, duplicate, and inclusive compounds. Due to the focus on designing small-molecule drugs, compounds with molecular weights greater than 600 were deleted from the dataset. In the original dataset, the number of non-agonist samples was significantly larger than that of agonist samples, which could result in the model being biased towards the majority class and performing poorly on the minority class. To address the class imbalance issue, undersampling was performed by randomly selecting an equal number of non-agonist samples as the agonist samples.

After completing all preprocessing steps, the chemical space of the dataset was examined. The molecular weight of the compounds in both datasets spanned from 100 to 600 Da, and the logP values were mainly distributed between −2 and 6. This indicates good chemical diversity, coverage, and balanced distribution (Supplementary Figure S1), meeting the requirements for drug development research. The chiral simplified molecular input line entry system (SMILES) of compounds was obtained using RDKit, and various molecular descriptors were generated using Deepchem, including molecular fingerprints—E2048, E1024, PUB, MACCS—as well as the featurizer of general graph convolution networks for molecules. To reduce redundant features, columns with zero variance were removed (Table 1). The molecular descriptors were used as features, and agonist (1) or non-agonist (0) labels as target values to build machine learning models.

### 4.2. Machine Learning Model Algorithms

Agonist prediction is a binary classification scenario. The algorithms employed in this study include ensemble learning (RF, LGBM from the Scikit-learn 1.5.1) and deep learning (GCN, GAT, MPNN, and AFP from the DeepChem 2.8.0). Decision trees are good at solving classification problems, and their ensemble patterns are categorized into two types: bagging and boosting. RF [46], one of the most classical bagging models, randomly samples partial data and features for training each tree, and performs equal-weight voting on the results. LGBM [47] is a boosting framework that learns and improves from each training iteration to obtain better learners through successive refinement. Additionally, graph representation has recently emerged as a frontier. Compared with molecular fingerprints, graph notation encodes more structural information [48]. Therefore, in addition to relatively classical deep learning models like GCN [49], GAT [50], and MPNN [51], graph-based neural networks suitable for drug discovery, such as AttentiveFP (AFP), have also emerged accordingly [52]. In this study, ensemble models were trained using various molecular fingerprints, while neural network models were trained with the featurizer of general graph convolution networks for molecules (abbreviated as Graph). The naming scheme follows the format [Target]_[Algorithm]_[Feature] (e.g., AHR_RF_E2048, NRF2_GCN_Graph). A complete list of model names is provided in Supplementary Table S1.

### 4.3. Cross-Validation and Hyperparameter Search

The dataset was stratified by class and randomly split into training, validation, and test sets at a ratio of 0.64, 0.16, and 0.2, respectively. The training and validation sets were used for hyperparameter optimization by averaging the results of 5-fold cross-validation, while the test set was employed to assess the predictive performance and generalization ability of the models. The hyperparameters of the ensemble models were automatically tuned using the Optuna 3.5.0 of Python 3.10.5, while parameter selection for the deep learning models was performed via grid search. Detailed information on the parameters is provided in Supplementary Tables S2 and S3.

### 4.4. Model Evaluation

For binary classification models, *PR*, *RE*, *ACC*, and *F*_1_ are typical indicators [53], which were integrated using the macro-averaging method. In addition, *AUC* was used to evaluate the classification performance of the models. For agonist classification, the threshold may not necessarily be 0.5, and the *AUC* is unaffected by the specific threshold, thus providing a comprehensive assessment of model performance. The formula is as follows:

(1) $$PR=\frac{1}{n}\sum_{i=1}^{n}\frac{{TP}_{i}}{{TP}_{i}+{FP}_{i}}$$

(2) $$RE=\frac{1}{n}\sum_{i=1}^{n}\frac{{TP}_{i}}{{TP}_{i}+{FN}_{i}}$$

(3) $$ACC=\frac{\sum_{i=1}^{n}{TP}_{i}}{m}$$

(4) $$F_{1}=2\times \frac{Precision\times Recall}{Precision+Recall}$$

where true positives (*TP*) refer to samples for which both the true label and the predicted label are positive; false positives (*FP*) refer to samples with a negative true label but a positive predicted label; and false negatives (*FN*) refer to samples with a positive true label but a negative predicted label.

### 4.5. Feature Importance Assessment

To gain insights into the structural differences between agonists and non-agonists from the model, a feature importance analysis was conducted on the best-performing model. The SHAP interpretable machine learning library can calculate the SHAP values for each sample and each feature, thereby analyzing the direction in which features influence the model’s prediction results. It is currently a widely used analysis tool [54]. Subsequently, RDKit was used to visualize the most important molecular fingerprints to further analyze the relationship between structure and activation.

### 4.6. Screening of Natural Compound Libraries

The natural compound library is from https://coconut.naturalproducts.net/ (accessed on 8 January 2025) [55], which contains 695142 compounds. First, duplicate values and rows with empty “name” were removed. Second, rows with all null values in ‘np_classifier_class’, ‘np_classifier_superclass’, and ‘np_classifier_pathway’ were deleted, as these indicate unknown compound categories. In accordance with the calculation criteria of the COCONUT website, compounds with np-likeness ≤ 0 were removed. Considering the chemical characteristics of drugs, natural compounds with a molecular weight < 500, logP between 0 and 5, hydrogen bond acceptors < 10, hydrogen bond donors < 5, and a CAS number were selected, totaling 17,159. The optimal-performing models were used for screening to obtain AHR/NRF2 dual agonists.

### 4.7. Cell Culture and Reagents

The HaCaT cell line was originally purchased from the National Collection of Authenticated Cell Cultures (Beijing, China). Cells were grown in MEM medium (Gibco, Grand Island, NY, USA) supplemented with 10% fetal bovine serum (FBS) (Corning, New York, NY, USA) and 1% penicillin/streptomycin (Gibco, Grand Island, NY, USA) at 37 °C in a humidified atmosphere of 5% CO_2_. Indirubin was purchased from MedChemExpress (Monmouth Junction, NJ, USA). Imperatorin, isoimperatorin, 3′-O-Methylbatatasin III, and bergapten were purchased from Targetmol (Boston, MA, USA). TBHQ was purchased from Solarbio (Beijing, China).

### 4.8. Cell Viability Assay

Cell viability was determined using the Cell Counting Kit-8 (CCK-8) assay and was done according to the manufacturer’s instructions (Sangon, Shanghai, China). Briefly, HaCaT cells were seeded in 96-well plates (2 × 10^4^ cells/well) and allowed to adhere overnight. Then, cells were treated with different natural compounds for 24 h. A total of 10 μL of CCK-8 reagent was added to each well, and cells were incubated at 37 °C for 1.5 h. The absorbance was measured at 450 nm using a multifunctional enzyme marker (Tecan, Männedorf, Switzerland). The asorbance of cells in the control group was regarded as 100% cell survival. All experiments were carried out three times in four parallel wells.

### 4.9. Dual Luciferase Reporter Gene Assay

Cells were first seeded in 96-well plates at a density of 2 × 10^4^ cells per well and cultured overnight to achieve complete adhesion; after adhesion, the cells were transfected with pGL4.43 [luc2P/XRE/Hygro] Vector (Promega, Madison, WI, USA) or pGL4.37 [luc2P/ARE/Hygro] Vector (Promega, Madison, WI, USA) and pRL-TK Vector (Promega, Madison, WI, USA) using Lipofectamine^®^ LTX & PLUS™ Reagent (Invitrogen, Carlsbad, CA, USA) according to the manufacturer’s instructions. Twenty-four hours later, cells were treated with different natural compounds. After 24 h, the luminescence was measured in a Promega GloMax-Multi microplate reader (Promega, Madison, WI, USA) using the Dual Luciferase Reporter Assay System (Promega, Madison, WI, USA). All experiments were carried out three times in four parallel wells.

### 4.10. Statistics

All experimental results are shown as the means ± SEMs. Statistical analyses were performed using GraphPad Prism version 9.0 (GraphPad Software, San Diego, CA, USA). Statistical significance among the different groups was tested by one-way analysis of variance, and *p* < 0.05 indicated statistical significance.

## 5. Conclusions

This study established a machine learning model for predicting dual AHR/NRF2 agonists, which showed good classification performance. Based on this model, we further identified key molecular features of dual agonists, namely a low fraction of sp^3^-hybridized carbons, moderate hydrophobicity, limited alkyl chains, and highly conjugated structures. Utilizing the established model, 1011 potential dual agonists of AHR/NRF2 from a natural compound library were screened for reference. For validation, Indirubin, imperatorin and 3′-O-Methylbutastatin III were first discovered as AHR/NRF2 dual agonists in HaCaT cells. Overall, this study facilitates and supports the screening of AD therapeutics. However, as AHR and NRF2 represent key metabolic pathways listed in TOX21, further investigation into their ligand characteristics is warranted to better leverage them as disease targets.

## Figures and Tables

**Table 1:** The lengths of molecular fingerprints used in this work.

Molecular Fingerprints	AHR		NRF2	
	Length (Bits)	Length After FS (Bits)	Length (Bits)	Length After FS (Bits)
E2048	2048	2048	2048	2040
E1024	1024	1024	1024	1024
MACCS	167	153	167	154
PUB	881	633	881	616

Note: FS stands for feature selection.

## Data Availability

The original contributions presented in this study are included in the article/Supplementary Materials. Further inquiries can be directed to the corresponding author.

## Supplementary Materials

The following supporting information can be downloaded at: https://www.mdpi.com/article/10.3390/ijms27083530/s1.

## Abbreviations

The following abbreviations are used in this manuscript:

AHR	Aryl Hydrocarbon Receptor
NRF2	Nuclear Factor Erythroid 2-Related Factor 2
NQO1	NAD(P)H Quinone Dehydrogenase 1
HO-1	Heme Oxygenase-1
GST	Glutathione S-Transferase
CAT	Catalase
SOD	Superoxide Dismutase
CYP1A1	Cytochrome P450 Family 1 Subfamily A Member 1
IL-4, 8, 13, 33	Interleukin-4, 8, 13, 33
FLG	Filaggrin
STAT6	Signal Transducer and Activator of Transcription 6
GABAA	Gamma-Aminobutyric Acid Type A Receptor
GPCR	G Protein-Coupled Receptor
PPAR-δ	Peroxisome Proliferator-Activated Receptor Delta
SMILES	Simplified Molecular Input Line Entry System
ECFP4	Extended-Connectivity Fingerprint with a diameter of 4
MACCS	Molecular ACCess System keys
NOX2	Nicotinamide Adenine Dinucleotide Phosphate Oxidase 2
