# Supplementary material for: AHR/NRF2 Dual Agonist Prediction and Natural Compound Screening Based on Machine Learning: A New Strategy for the Treatment of Atopic Dermatitis
Source: Int J Mol Sci. 2026 Apr 15;27(8):3530. doi: 10.3390/ijms27083530 (PMC13115769; doi:10.3390/ijms27083530)
Supplement: Supplementary file 1 [file ijms-27-03530-s001.zip › Dataset S1 Predicted dual AHR&NRF2 agnoists.pdf]

Potential AHR/NRF2 agonists screened from natural products library

identifier	canonical_smiles	name
CNP0036440.0	CC(C)c1cccc(C(C)C)c1O	propofol
CNP0050375.0	C1=Cc2ccccc2OC1	2H-Chromene
CNP0076017.0	OC1Cc2ccccc2OC1c1ccccc1	Flavan-3-ol
CNP0103410.0	COc1ccc(-c2cc(=O)c3c(OC)cc(OC)c(OC)c3o2)cc1OC	Isosinensetin
CNP0112221.0	O=c1cc(-c2ccccc2)oc2c(O)c(O)ccc12	7,8-dihydroxyflavone
CNP0119714.0	O=c1cc(-c2ccccc2)oc2ccc(O)cc12	6-Hydroxyflavone
CNP0120850.0	COc1ccc(-c2coc3cc(O)cc(O)c3c2=O)cc1O	Pratensein
CNP0123354.3	c1cc2c(cc1[C@H]1OC[C@H]3[C@@H]1CO[C@@H]3c1ccc3c(c1)OCO3)OCO2	sesamin
CNP0128543.1	C1=CC[C@@H](c2cccnc2)NC1	ANATABINE
CNP0128543.2	C1=CC[C@H](c2cccnc2)NC1	(R)-(+)-Anatabine
CNP0137967.0	COc1ccc(-c2oc3cc(O)cc(O)c3c(=O)c2O)cc1O	Tamarixetin
CNP0139758.0	COc1ccc2c(OC)c3ccoc3nc2c1OC	Skimmianine
CNP0143972.0	Oc1ccccc1O	pyrocatechol
CNP0144713.0	O=c1cc(-c2ccccc2)oc2cc(O)c(O)cc12	6,7-dihydroxyflavone
CNP0145919.0	COc1cc(O)c2c(=O)cc(-c3ccc(O)cc3)oc2c1	Genkwanin
CNP0155660.0	COC(=O)c1c2c(c3ccccc3c1O)OC(C)(C)C=C2	Mollugin
CNP0166877.0	COc1ccc(-c2cc(=O)c3c(O)c(OC)c(OC)c(OC)c3o2)cc1OC	Demethylnobiletin
CNP0167923.0	O=c1cc(-c2ccc(Br)cc2)oc2ccccc12	4'-bromoflavone
CNP0170325.0	COc1cc2oc(-c3ccc(O)c(O)c3)c(OC)c(=O)c2c(O)c1OC	Chrysosplenol D
CNP0172176.0	COc1ccc(-c2cc(=O)c3c(OC)c(OC)c(OC)cc3o2)cc1OC	Sinensetin
CNP0178431.2	Oc1cc2c(cc1[C@H]1OC[C@H]3[C@@H]1CO[C@@H]3c1ccc3c(c1)OCO3)OCO2	Sesaminol

CNP0180331.0	COc1cc(-c2oc3cc(OC)c(OC)c(O)c3c(=O)c2OC)ccc1O	Chrysosplenetin
CNP0184442.0	COc1cc2ccc(=O)oc2cc1O	scopoletin
CNP0184539.0	Cc1cc(O)c2c(c1)C(=O)c1cccc(O)c1C2=O	Chrysophanol
CNP0187563.0	CC(C)=CCOc1c2ccoc2cc2oc(=O)ccc12	Isoimperatorin
CNP0187845.0	C=CCc1ccc(O)c(-c2ccc(O)c(CC=C)c2)c1	Honokiol
CNP0189885.0	O=c1oc2cc(O)ccc2c2cc(O)c(O)cc12	Urolithin C
CNP0197797.0	OCc1ccc(-c2nccc3c2[nH]c2ccccc23)o1	Perlolyrine
CNP0202297.1	COc1cc([C@H]2O[C@@H](c3ccc(O)c(OC)c3)[C@H](C)[C@@H]2C)ccc1O	Nectandrin B
CNP0205963.0	O=c1ccc2ccc(O)c(O)c2o1	daphnetin
CNP0206775.0	Cc1cccc2c1ccc1c3occ(C)c3oc(=O)c21	Tanshinlactone
CNP0209864.0	COc1ccc(-c2cc(=O)c3c(OC)c(OC)c(OC)c(OC)c3o2)cc1OC	Nobiletin
CNP0212412.1	C=C[C@H](c1ccccc1O)c1cc(O)c(OC)cc1OC	Latifolin
CNP0214195.0	Cc1cc2c(cc1O)C(=O)c1ccccc1C2=O	2-Hydroxy-3-methylanthraquinone
CNP0223104.0	Oc1ccc2c(c1)OCO2	Sesamol
CNP0223782.0	COc1cc(-c2cc(=O)c3c(O)cc(O)cc3o2)ccc1O	Chrysoeriol
CNP0226937.1	O=C1c2c(O)cc(O)cc2O[C@H](c2ccccc2)[C@H]1O	Pinobanksin
CNP0228309.0	C=CCc1cccc(O)c1O	Allylpyrocatechol
CNP0228777.0	COc1ccc(-c2cc(=O)c3c(O)c(OC)c(OC)cc3o2)cc1	Salvigenin
CNP0229171.0	COc1cccc(O)c1-c1cc(=O)c2c(O)c(OC)c(OC)c(OC)c2o1	Skullcapflavone II
CNP0230702.0	O=Cc1ccc(O)cc1	4-hydroxybenzaldehyde
CNP0231615.0	COc1cc(O)c2c(=O)c(-c3ccc(O)cc3)coc2c1	Prunetin
CNP0232100.0	O=C1c2ccccc2C(=O)c2c(O)c(O)cc(O)c21	PURPURIN

CNP0233225.0	O=c1oc2cc(O)ccc2c2ccccc12	urolithin B
CNP0234330.0	COc1cc(-c2cc(=O)c3c(O)c(OC)c(O)c(OC)c3o2)ccc1O	SUDACHITIN
CNP0236338.0	O=c1c(O)c(-c2ccccc2)oc2cc(O)cc(O)c12	Galangin
CNP0237570.0	COc1cc(O)c2c(=O)c(O)c(-c3ccc(O)c(OC)c3)oc2c1	Rhamnazin
CNP0249075.0	O=c1c(O)c(-c2ccc(O)cc2)oc2ccccc12	3,4'-DIHYDROXYFLAVONE
CNP0263163.0	O=c1cc(-c2ccccc2)oc2cc(O)ccc12	7-Hydroxyflavone
CNP0267409.0	COc1cc(O)c2c(=O)cc(-c3ccccc3)oc2c1OC	Moslosooflavone
CNP0268131.0	O=c1cc(-c2ccc(O)c(O)c2)oc2ccccc12	3',4'-Dihydroxyflavone
CNP0270767.0	CC(C)=CCOc1c2occc2cc2ccc(=O)oc12	IMPERATORIN
CNP0276366.0	O=c1c(O)c(-c2ccc(O)c(O)c2)oc2cc(O)ccc12	Fisetin
CNP0283350.0	COc1ccc2c(c1)OC(C)(C)C=C2	Precocene I
CNP0284444.0	CC(C)=CCc1cc(-c2coc3cc(O)ccc3c2=O)ccc1O	Neobavaisoflavone
CNP0286100.0	COc1cc2ccc(=O)oc2cc1OC	Scoparone
CNP0287011.0	COc1ccc2c(c1)[nH]c1c(C)nccc12	HARMINE
CNP0290456.0	O=c1cc(-c2ccc(O)cc2)oc2cc(O)c(O)c(O)c12	Scutellarein
CNP0291283.0	O=c1ccc2ccc(O)cc2o1	Umbelliferone
CNP0295329.0	COc1ccc2c(=O)cc(-c3ccccc3)oc2c1	7-Methoxyflavone
CNP0295922.0	COc1cc2ccc(=O)oc2c(O)c1O	Fraxetin
CNP0306254.0	COc1cc(-c2[o+]c3cc(O)cc(O)c3cc2O)cc(OC)c1O	Malvidin
CNP0308187.0	Oc1cccc(O)c1O	pyrogallol
CNP0310279.0	O=c1c(O)c(-c2ccc(O)cc2O)oc2cc(O)cc(O)c12	morin
CNP0313419.0	O=c1c(-c2ccccc2)coc2ccccc12	Isoflavone

CNP0320767.0	O=C1Nc2ccccc2C1=O	Isatin
CNP0322936.0	O=c1c(-c2ccc(O)cc2)coc2cc(O)c(O)c(O)c12	6-Hydroxygenistein
CNP0323885.0	O=c1ccoc2cc(O)cc(O)c12	5,7-Dihydroxychromone
CNP0325061.0	COc1cc2oc(-c3ccccc3)cc(=O)c2c(O)c1O	Negletein
CNP0345595.0	Nc1ccc2oc(-c3ccccc3)cc(=O)c2c1	6-Aminoflavone
CNP0348920.0	COc1ccc(-c2oc3cc(OC)c(OC)c(O)c3c(=O)c2OC)cc1OC	Artemetin
CNP0357819.0	C=Cc1ccc(O)c(OC)c1	2-Methoxy-4-vinylphenol
CNP0366241.1	Oc1ccc2c(c1)OC[C@H]1c3cc4c(cc3O[C@@H]21)OCO4	(-)-Maackiain
CNP0386988.0	O=c1ccc2cc(O)c(O)cc2o1	Esculetin
CNP0391742.0	COc1cc2ccc(=O)oc2c(OC)c1O	Isofraxidin
CNP0392086.0	COc1cc(O)c2c(=O)c(OC)c(-c3ccc(O)c(OC)c3)oc2c1	Pachypodol
CNP0394762.0	COc1cc(O)c2c(=O)cc(-c3ccc(O)c(O)c3)oc2c1	Hydroxygenkwanin
CNP0401169.0	CC(C)=CCc1cc(-c2oc3cc(O)cc(O)c3c(=O)c2O)ccc1O	Isolicoflavonol
CNP0405816.0	COc1ccc(-c2cc(=O)c3c(O)c(OC)c(OC)cc3o2)cc1O	Eupatorin
CNP0406562.0	O=C(O)c1cc2c([nH]c3ccccc32)c(-c2ccc(CO)o2)n1	Flazin
CNP0414137.0	Oc1cccc(O)c1	resorcinol
CNP0468383.0	Oc1ccc(C2CNCCc3c2cc(O)c(O)c3Cl)cc1	fenoldopam
CNP0485082.0	COc1cc(-c2[o+]c3cc(O)cc(O)c3cc2O)cc(O)c1O	Petunidin
CNP0558669.0	C1=Cc2ccccc2NN=C1	Benzodiazepine
CNP0583275.0	COc1cc(-c2[o+]c3cc(O)cc(OC)c3cc2O)cc(O)c1O	Europinidin
CNP0604242.0	O=C1Oc2ccccc2Oc2ccccc21	Depsidone
CNP0027717.0	Cc1c2ccccc2c(C)c2ccccc12	9,10-DIMETHYLANTHRACENE

CNP0028775.0	Cc1cc(O)cc2oc(-c3ccccc3)cc(=O)c12	7-Hydroxy-5-methylflavone
CNP0029083.0	Cc1cc(OP(=O)(O)O)c2ccccc2c1OP(=O)(O)O	Menadiol diphosphate
CNP0030526.0	O=c1oc2cc(O)ccc2c(-c2ccccc2)c1-c1ccccc1	3,4-DIPHENYL-7-HYDROXYCOUMARIN
CNP0039859.0	CC(=O)Oc1c(-c2ccccc2)oc2ccccc2c1=O	3-ACETOXYFLAVONE
CNP0040242.0	COc1cccc(-c2oc3ccc(OC)cc3c(=O)c2OC)c1	3,6,3'-TRIMETHOXYFLAVONE
CNP0042389.0	O=c1oc2ccccc2c(O)c1C1CCCc2ccccc21	COUMATETRALYL
CNP0042735.0	C1=Cc2ccccc2Cc2ccccc21	5H-Dibenzo[a,d]cycloheptene
CNP0045596.0	COc1ccc2c(=O)cc(-c3cc(OC)c(OC)c(OC)c3)oc2c1	7,3',4',5'-tetramethoxyflavone
CNP0047791.0	Nc1ccc2c(c1)C(=O)c1ccccc1C2=O	2-AMINOANTHRAQUINONE
CNP0047972.0	Cc1cccc2c(=O)cc(-c3ccccc3)oc12	8-METHYLFLAVONE
CNP0049197.0	c1cc2ccc1CCc1ccc(cc1)CC2	[2.2]Paracyclophane
CNP0050486.0	COc1ccccc1-c1c(C)oc2cc(OC(C)=O)ccc2c1=O	3-(2-methoxyphenyl)-2-methyl-4-oxo-4H-chromen-7-yl acetate
CNP0060813.0	COc1cc(O)c(C(=O)C=Cc2ccccc2Cl)c(OC)c1	2-CHLORO-4',6'-DIMETHOXY-2'-HYDROXYCHALCONE
CNP0062439.0	Oc1ccc2oc3c(Cl)c(Cl)ccc3c2c1	8-Hydroxy-3,4-dichlorodibenzofuran
CNP0064627.0	COc1cc2occ(-c3ccccc3)c(=O)c2cc1OC	6,7-Dimethoxyisoflavone
CNP0066279.0	Cc1ccc2oc(-c3ccccc3)c(O)c(=O)c2c1	6-Methylflavonol
CNP0069439.0	O=C1C=CC(=O)c2cc3ccccc3cc21	1,4-Anthraquinone
CNP0076061.0	CC1=C(O)c2ccc3c(C)cccc3c2C(=O)C1=O	Danshenxinkun C
CNP0077190.0	OC1c2ccccc2Oc2ccccc21	9-Hydroxyxanthene
CNP0078034.0	S=c1c2ccccc2oc2ccccc12	Xanthene-9-thione
CNP0078216.0	Oc1cccc2[nH]c3ccccc3c12	4-Hydroxycarbazole
CNP0079543.0	O=C1c2cc3ccccc3cc2C(=O)c2cc3ccccc3cc21	6,13-Pentacenequinone

CNP0079764.0	COc1ccc(-c2ccc3cccc4c3c2C(=O)C=C4)cc1	9-(4-methoxyphenyl)phenalen-1-one
CNP0079922.0	Cc1ccnc2c1C(=O)c1ccccc1-2	Onychine
CNP0083766.0	Oc1ccc2cc3c4c(ccc5cccc(c54)C4OC34)c2c1	9-Hydroxybenzo[a]pyrene-4,5-oxide
CNP0084711.1	O=C(O)[C@H]1O[C@@H](Oc2ccc3c(c2)oc(=O)c2ccccc23)[C@H](O)[C@@H](O)[C@@H]1O	Urolithin B 3-O-glucuronide
CNP0088497.0	COc1cc(C=O)cc2c1[nH]c1ccccc12	Murrayanine
CNP0095267.0	c1ccc(-c2cccc3c2[nH]c2ccccc23)cc1	1-phenyl-9h-carbazole
CNP0102563.0	COc1c(C(=O)c2ccc3c(c2)OCO3)oc2c1ccc1occc12	Derriobtusone B
CNP0103144.0	Cc1cc(O)c2c3c(ccc2c1)C(=O)c1c(O)cccc1C3=O	Tetrangulol
CNP0103237.0	COc1ccc2c(c1)C(=O)c1c(O)c(OC)c(OC)c3ccnc-2c13	Dauriporphinoline
CNP0103766.0	COc1cc2c(c(OC)c1)-c1c(O)cccc1CC2	Loroglossol
CNP0104431.0	COc1cc2ccc3cc(OC)c(OC)cc3c2cc1OC	2,3,6,7-tetramethoxyphenanthrene
CNP0104616.1	COc1c2c(nc3occc13)[C@@](CC=C(C)C)(OC)[C@H](O)CC2	Haplophyllidine
CNP0104737.0	CC#Cc1ccc(-c2ccc(C(C)=O)s2)s1	Arctinone b
CNP0105066.0	COc1c2c(cc3oc(=O)ccc13)OC(C)(C)C=C2	Xanthoxyletin
CNP0106089.0	COc1cc(C=Cc2ccc(O)cc2)cc(OC)c1	4-[2-(3,5-dimethoxyphenyl)ethenyl]phenol
CNP0106338.0	O=c1cc(-c2ccc(O)cc2)oc2ccccc12	4'-Hydroxyflavone
CNP0106770.0	COc1ccc(C=Cc2cc(OC)c(OC)c(OC)c2)cc1O	2-methoxy-5-[2-(3,4,5-trimethoxyphenyl)ethenyl]phenol
CNP0106892.0	COc1c2ccoc2nc2c(OC)cccc12	gamma-Fagarine
CNP0107191.0	COc1ccc(-c2cc(=O)c3cc(OC)ccc3o2)cc1	6,4'-DIMETHOXYFLAVONE
CNP0107474.0	O=C1NCc2c1c1c3ccccc3[nH]c1c1[nH]c3ccccc3c21	K-252c
CNP0107588.0	COc1cc2c(oc3ccccc32)c(OC)c1O	Eriobofuran
CNP0107618.0	COc1cc2oc3ccccc3c(=O)c2cc1O	2-hydroxy-3-methoxyxanthone

CNP0107704.0	COc1c2c(cc3ccc(=O)oc13)C=CC(C)(C)O2	Luvangetin
CNP0107788.0	c1ccc2c(c1)Cc1ccc3ccccc3c1-2	7H-Benzo[c]fluorene
CNP0107987.0	COc1c2occc2c(OCC=C(C)C)c2ccc(=O)oc12	Cnidilin
CNP0108377.0	O=Cc1ccc2[nH]c3ccccc3c2c1	9H-Carbazole-3-carbaldehyde
CNP0108554.0	COc1ccc(-c2coc3cc(OC)c(OC)cc3c2=O)cc1	4',6,7-Trimethoxyisoflavone
CNP0108804.0	O=C1c2ccccc2-c2nccc3ccnc1c23	Sampangine
CNP0109029.0	O=C1Nc2cc(Br)ccc2C1=O	6-bromoisatin
CNP0109076.0	COc1c(O)cc(OC)c2c(=O)cc(-c3ccccc3)oc12	7-Hydroxy-5,8-Dimethoxyflavone
CNP0109364.0	CC(=O)c1nccc2c1[nH]c1ccccc12	1-Acetyl-beta-carboline
CNP0109775.0	COc1cccc2c1C(=O)c1c(OC)cccc1C2=O	1,8-Dimethoxyanthraquinone
CNP0109854.0	CC(C)c1c(O)cc(C=Cc2ccccc2)cc1O	5-(2-phenylethenyl)-2-propan-2-ylbenzene-1,3-diol
CNP0110412.0	COc1cc2c(cc1-c1coc3cc4occc4cc3c1=O)OCO2	Dehydroneotenone
CNP0111764.0	COc1ccc(OC)c2c1C(=O)c1ccccc1C2=O	1,4-Dimethoxyanthraquinone
CNP0111804.0	O=C1c2ccccc2C(=O)c2c1ccc1c2OCO1	Morindaparvin A
CNP0112288.0	Cc1cc(O)c2c(c1)C(=O)c1ccccc1C2=O	PACHYBASIN
CNP0112856.0	O=c1cc(-c2cccc(O)c2)oc2ccccc12	3'-hydroxyflavone
CNP0113186.0	Oc1c2ccccc2cc2ccccc12	anthracen-9-ol
CNP0113267.0	c1ccc2c(c1)-c1nccc3ccnc-2c13	Eupolauridine
CNP0113295.0	COc1c(O)ccc2c1C(=O)c1ccccc1C2=O	Alizarin 1-methyl ether
CNP0113505.0	COc1cc(OC)c2c(-c3ccccc3)cc(=O)oc2c1	5,7-dimethoxy-4-phenylchromen-2-one
CNP0113847.0	Cc1cc(CO)c2c(=O)c3c(O)cccc3oc2c1	8-hydroxy-1-(hydroxymethyl)-3-methylxanthen-9-one
CNP0114280.0	COc1cc(OC)c2c(=O)c3cc(OC)ccc3oc2c1	1,3,7-Trimethoxyxanthone

CNP0115082.0	COc1c2cc3c(cc2nc2occc12)OCO3	Maculine
CNP0115549.0	C=C(C)c1cc2c(ccc3ccc(=O)oc32)o1	Oroselone
CNP0116487.0	COc1c(C)c(C=O)c2[nH]c3ccccc3c2c1O	Carbazomycin E
CNP0117304.0	O=C1Oc2ccccc2C1=Cc1ccccc1	benzalcoumaranone
CNP0121723.0	COc1cc2oc(=O)ccc2cc1-c1c(OC)ccc2ccc(=O)oc12	Matsukaze-lactone
CNP0122909.0	COc1ccc2oc(-c3ccccc3)cc(=O)c2c1	6-Methoxyflavone
CNP0123271.0	COc1c(OC)c2cc3ccoc3c(OC)c2oc1=O	Isohalfordin
CNP0123708.0	c1ccc2cc3c(cc2c1)oc1ccccc13	Benzo[b]naphtho[2,3-d]furan
CNP0124451.0	COc1ccc2c(c1)OCc1c-2oc2cc3c(cc12)OCO3	Anhydropisatin
CNP0124971.0	CC1(C)C=Cc2c(cc(O)cc2-c2cc3ccc(O)cc3o2)O1	Moracin E
CNP0128288.0	COc1cccc2c1C(=O)c1ccc3cc(C)cc(O)c3c1C2=O	8-O-Methyltetrangulol
CNP0128687.0	COc1c2ccoc2nc2c3c(ccc12)OCO3	Kokusagine
CNP0129360.0	O=Cc1c[nH]c2cc(Br)c(Br)cc12	5,6-Dibromo-1H-indole-3-carbaldehyde
CNP0130117.0	c1cnc2c(C3=NCCc4c3[nH]c3ccccc43)cccc2c1	Komarovidine
CNP0130280.0	C=C(C)c1cc2c(=O)oc3cccc(C)c3c2o1	Pterophyllin 2
CNP0130683.0	COc1ccc2c(c1)C(=O)c1c(OC)c(OC)cc3ccnc-2c13	Menisporphine
CNP0133041.0	COc1ccc(-c2cc(=O)c3c(OC)c(OC)c(OC)cc3o2)cc1	Scutellarein tetramethyl ether
CNP0133365.0	CC(=O)c1c(O)ccc2c(=O)c(-c3ccccc3)c(C)oc12	Glyzarin
CNP0134985.0	Cc1cccc2c1C(=O)c1ccccc1C2=O	1-Methylanthraquinone
CNP0138948.0	COc1c(CC=C(C)C)c(O)cc2c1-c1oc3cc(O)ccc3c1CO2	Glyurallin A
CNP0139900.0	COc1c2ccoc2nc2c(O)c3c(c(CC=C(C)C)c12)OCO3	Tecleaverdoornine
CNP0140763.0	C=C(C)c1cc2c(o1)C(=O)c1ccccc1C2=O	2-isopropenylnaphtho[2,3-b]furan-4,9-dione

CNP0141603.0	COc1cc2ccnc3c2c(c1OC)-c1ccccc1C3=O	Lysicamine
CNP0141767.0	O=C1c2ccccc2-c2ccccc21	9-Fluorenone
CNP0142128.0	COc1c(C)c(C)c2[nH]c3ccccc3c2c1O	Carbazomycin B
CNP0142284.0	COc1cc2ccnc3c2c(c1O)N(C)C=C3	Isoaaptamine
CNP0142509.0	Oc1cccc2c1ccc1c3ccccc3ccc21	1-Hydroxychrysene
CNP0142627.0	O=c1c(-c2ccccc2)coc2cc(O)ccc12	7-Hydroxyisoflavone
CNP0142870.0	COc1ccc2c(c1O)C(=O)c1ccccc1C2=O	1-Hydroxy-2-methoxyanthraquinone
CNP0143162.0	COc1ccc2c(c1)C(=O)c1cc(OC)cc3ccnc-2c13	Bianfugecine
CNP0146636.0	O=c1[nH]c2ccccc2c(-c2ccccc2)c1O	Viridicatin
CNP0146770.0	Oc1cccc2nc3ccccc3nc12	1-Hydroxyphenazine
CNP0147138.0	COc1cc2c(cc1-c1coc3cc(O)ccc3c1=O)OCO2	Maxima isoflavone G
CNP0147310.0	COc1c2ccc(-c3ccccc3)oc-2cc(=O)c1C	Dracorhodin
CNP0147647.0	COc1ccc(C(=O)c2nccc3cc4c(cc23)OCO4)c(O)c1OC	Rugosinone
CNP0147935.0	CC(C)=CCc1c2c(c3[nH]c4c(O)cccc4c(=O)c3c1O)C=CC(C)(C)O2	Atalaphillinine
CNP0148936.0	Cc1cc2c([nH]c3cc(O)ccc32)c2c1OC(C)(C)C=C2	Murrayamine A
CNP0149125.0	O=c1cc(-c2ccccc2)oc2ccc(O)c(O)c12	5,6-DIHYDROXYFLAVONE
CNP0149238.0	COc1c(C(=O)CC(=O)c2ccc3c(c2)OCO3)ccc2occc12	Ovalitenone
CNP0149826.0	CC(=O)c1nc(C(=O)O)cc2c1[nH]c1ccccc12	9H-Pyrido(3,4-b)indole-3-carboxylic acid, 1-acetyl-
CNP0150206.0	C=C(C)c1cc2cc(C(C)=O)ccc2o1	DEHYDROTREMETONE
CNP0150848.0	COc1cc2nccc3c4ccccc4n(c1=O)c23	5-Methoxycanthin-6-one
CNP0151162.0	c1ccc2c(c1)ncc1cc3c(cc12)OCO3	Trisphaeridine
CNP0151894.0	COc1ccc2oc3cc(OC)c(OC)c(OC)c3c(=O)c2c1	1,2,3,7-Tetramethoxyxanthone

CNP0152796.0	O=C1c2ccccc2-c2c1oc1ccccc1c2=O	Wrightiadione
CNP0152956.0	COc1cc2cc(-c3cc(O)cc(O)c3)oc2cc1OC	Moracin F
CNP0153902.0	COc1cc(C)cc2c1[nH]c1ccccc12	1-Methoxy-3-methyl-9H-carbazole
CNP0154296.0	O=c1oc2cc3occc3cc2c2oc3cc4c(cc3c12)OCO4	Erosnin
CNP0154400.0	COc1ccc2c(=O)c(OC)c(-c3ccc(OC)c(OC)c3)oc2c1	Fisetin tetramethyl ether
CNP0154512.0	O=C1C(O)=Cc2coc(=O)c3ccc(-c4ccccc4)c1c23	Lachnanthopyrone
CNP0155104.0	COc1ccc2c(c1OC)C(=O)c1nccc3cc4c(c-2c13)OCO4	Oxocrebanine
CNP0155175.0	CC(C)=CCc1ccc2c(=O)c3c(O)cccc3oc2c1O	Calophyllin B
CNP0155176.0	COc1cc2oc3cc(OC)c(OC)c(OC)c3c(=O)c2cc1OC	1,2,3,6,7-pentamethoxyxanthone
CNP0155310.0	O=c1cc(CCc2ccccc2)oc2ccccc12	2-(2-Phenylethyl)chromone
CNP0155456.0	COc1ccc(-c2cc(=O)c3ccc(OC)cc3o2)cc1	7,4'-Dimethoxyflavone
CNP0156051.0	COc1c(OC)c(OC)c2c(=O)cc(-c3ccccc3)oc2c1OC	5,6,7,8-Tetramethoxyflavone
CNP0156063.1	Nc1oc2ccccc2c1C(=O)Cc1ccccc1O[C@@H]1O[C@H](CO)[C@@H](O)[C@H](O)[C@H]1O	Acuminaminoside
CNP0159011.0	COc1c2ccccc2nc2occc12	Dictamnine
CNP0159182.0	O=c1c2c(O)cc(O)cc2oc2oc3ccccc3c12	Ayamenin B
CNP0159250.0	COc1cc2ccc(=O)oc2c2c1OC(C)(C)C=C2	Braylin
CNP0159483.0	COc1ccc2c(ccc3cc(OC)c(OC)cc32)c1	2,3,7-TRIMETHOXYPHENANTHRENE
CNP0159893.0	COc1c2c(cc3occ(-c4ccccc4O)c(=O)c13)OCO2	Betavulgarin
CNP0160760.0	COc1cc(OC)c2c(=O)c(OC)c(-c3ccccc3)oc2c1	3,5,7-Trimethoxyflavone
CNP0161333.0	COc1c(OC)c(OC)c2c(=O)cc(-c3ccc4c(c3)OCO4)oc2c1OC	Linderoflavone B
CNP0161387.0	Cc1ccc(-c2cc(=O)c3ccccc3o2)cc1	2-(4-methylphenyl)-4H-chromen-4-one
CNP0161928.0	COc1ccc(-c2cc(=O)c3c(OC)cc(OC)cc3o2)cc1	4',5,7-Trimethoxyflavone

CNP0162044.0	Oc1cc2c(cc1Br)[nH]c1c(C3=NCCC3)nccc12	Eudistomin P
CNP0162574.0	C=C(C)c1ccc(C)c2ccc(C=O)c-2c1	Lactaroviolin
CNP0163611.0	O=C1OCc2c1cc1cc3c(cc1c2-c1ccc2c(c1)OCO2)OCO3	JUSTICIDIN E
CNP0164112.0	O=C1CCCc2c1[nH]c1ccccc21	2,3,4,9-tetrahydro-1H-carbazol-1-one
CNP0164517.0	O=c1cc(-c2ccccc2O)oc2cccc(O)c12	5,2'-Dihydroxyflavone
CNP0165031.0	COc1ccc2c(=O)c(-c3cc(OC)c(OC)cc3OC)coc2c1	7,2',4',5'-tetramethoxyisoflavone
CNP0165314.0	COc1ccc(-c2cc(=O)c3c(OC)cc(OC)c(OC)c3o2)cc1	6-Demethoxytangeretin
CNP0165688.0	COc1ccc2c(=O)cc(-c3ccccc3)oc2c1O	8-hydroxy-7-methoxyflavone
CNP0166234.0	COc1cc2oc(-c3ccccc3)c(OC)c(=O)c2c(OC)c1OC	3,5,6,7-Tetramethoxyflavone
CNP0166366.0	Cc1c(C(=O)O)nc(-c2ccc3c(n2)C(=O)C(N)=CC3=O)c2[nH]c3ccccc3c12	Lavendamycin
CNP0167217.0	O=c1c(O)c(-c2ccccc2O)oc2ccccc12	3,2'-Dihydroxyflavone
CNP0168837.0	COc1cc2[nH]c3c(O)cccc3c(=O)c2c(O)c1CC=C(C)C	Buxifoliadine C
CNP0169650.0	COC1=CC(=O)c2ccc3cccc(O)c3c2C1=O	Cymbinodin A
CNP0170396.0	COc1ccc2oc3cccc(OC)c3c(=O)c2c1	1,7-dimethoxyxanthone
CNP0170758.0	COc1ccc2oc3c(O)cccc3c(=O)c2c1	5-Hydroxy-2-methoxyxanthone
CNP0171103.0	COc1cccc(-c2oc3cc(OC)ccc3c(=O)c2OC)c1	3,7,3'-TRIMETHOXYFLAVONE
CNP0171104.0	COc1cc2oc3ccc(O)cc3c(=O)c2c(OC)c1OC	Onjixanthone I
CNP0171440.0	COc1cc2oc(-c3ccccc3)cc(=O)c2cc1OC	6,7-dimethoxyflavone
CNP0171699.0	COc1cc(OC)c2c(c1)C(=O)c1ccccc1C2=O	1,3-dimethoxyanthraquinone
CNP0171752.0	O=C1c2ccc3c(c2-c2c4c(cc5ccnc1c25)OCO4)OCO3	Hernandonine
CNP0172609.0	Oc1cccc2c1ccc1ccccc12	1-Hydroxyphenanthrene
CNP0172996.0	COc1cc2c(c3oc(-c4ccccc4)cc(=O)c13)C=CC(C)(C)O2	Isopongaflavone

CNP0173741.1	COc1c2c(c(C)c3c(C)coc13)[C@@H](C)CC=C2	Cacalohastine
CNP0174586.0	Cc1cc(=O)[nH]c2c1C(=O)c1ccccc1C2=O	Marcanine A
CNP0175009.0	COc1ccc2[nH]c3c(c2c1)CCN=C3C	6-Methoxyharmalan
CNP0176301.0	COc1c(OC)c2c3c(nccc3c1OC)C(=O)c1ccccc1-2	Homomoschatoline
CNP0178863.0	COc1ccc(-c2cc(=O)c3ccc(OC)cc3o2)c(OC)c1	7,2',4'-Trimethoxyflavone
CNP0179261.0	COc1cc2cc3c(c(-c4ccc5c(c4)OCO5)c2cc1OC)C(=O)OC3	Justicidin B
CNP0179264.0	O=c1cc(-c2ccccc2)oc2cccc(O)c12	5-Hydroxyflavone
CNP0180186.0	O=C1C=CC2(Oc3cccc4cccc(c34)O2)c2cccc(O)c21	Palmarumycin CP(1)
CNP0181233.0	O=C1C2=C(COC=C2)C(=O)c2ccccc21	Pentalongin
CNP0183838.0	O=c1ccc2c(cc(O)c3c4ccccc4nnc23)o1	Necatorin
CNP0184198.0	COc1cc2c3c(c1)COc1cc(O)cc(c1-3)CC2	Flavidinin
CNP0184435.0	C=Cc1c(C)c(O)cc2ccc3c(C)c(O)ccc3c12	Dehydrojuncusol
CNP0185890.0	CC(C)(O)c1cc2c(o1)C(=O)c1ccccc1C2=O	AVICEQUINONE C
CNP0186804.0	COc1cc(OC)c2cc(-c3cc(O)cc(O)c3)oc2c1	Moracin A
CNP0187794.0	COc1cc(O)cc(-c2cc3c(O)cccc3o2)c1	Gnetifolin M
CNP0187828.0	COc1c2c(cc3occ(-c4ccc(O)cc4)c(=O)c13)OCO2	Irisolone
CNP0188163.0	COc1cccc(OC)c1-c1cc(=O)c2c(OC)c(OC)ccc2o1	Zapotin
CNP0188462.0	O=c1[nH]c(=O)c2c3c([nH]c4ccccc43)oc3[nH]c4ccccc4c3c1=2	Arcyroxepin A
CNP0189685.1	C[C@H](O)c1nccc2c1[nH]c1ccccc12	Cordysinin D
CNP0190243.0	COc1cc2c(cc1-c1coc3cc(OCC=C(C)C)ccc3c1=O)OCO2	Maxima isoflavone C
CNP0190905.0	O=C(c1ncc(-c2c[nH]c3cc(Br)ccc23)[nH]1)c1c[nH]c2cc(O)ccc12	Bromotopsentin
CNP0191008.0	C=CC(C)(C)c1cc2cc3ccoc3cc2oc1=O	Chalepensin

CNP0191326.0	COc1ccccc1-c1cc(=O)c2ccccc2o1	2'-Methoxyflavone
CNP0191983.0	C=Cc1ncc(OC)c2c1[nH]c1c(O)cccc12	Picrasidine I
CNP0192835.0	c1ccc2c(c1)[nH]c1c(C3=NCCC3)nccc12	Eudistomin I
CNP0192915.0	O=c1oc(-c2ccccc2)cc2ccccc12	3-Phenyl-1H-isochromen-1-one
CNP0193564.0	COc1ccccc1-c1coc2cc3c(c(OC)c2c1=O)OCO3	Tlatlancuayin
CNP0194441.0	COc1ccc2c(=O)c(-c3ccc(OC)c(OC)c3)coc2c1	7,3',4'-Trimethoxyisoflavone
CNP0194478.0	COc1cc2c3c(cc4ccccc4c3c1O)NC2=O	Piperolactam A
CNP0195314.0	COc1ccc2c(=O)cc(-c3ccccc3OC)oc2c1	7,2'-DIMETHOXYFLAVONE
CNP0195687.0	CC1(C)C=Cc2cc3ccc(=O)oc3cc2O1	Xanthyletin
CNP0198067.0	COc1ccc(-c2cc(=O)c3c(O)cccc3o2)cc1	5-Hydroxy-4'-methoxyflavone
CNP0200085.0	COc1cccc2cc(-c3cc(=O)oc4ccc(C)cc34)oc12	4-(7-methoxy-1-benzofuran-2-yl)-6-methyl-2H-chromen-2-one
CNP0202664.0	COc1cc2oc(=O)cc(-c3ccccc3)c2cc1O	Dalbergin
CNP0202672.0	COc1c(C)c(O)cc2c1C(=O)c1ccccc1C2=O	Rubiadin 1-methyl ether
CNP0202997.0	COC1=C(c2ccccc2)C(=O)C(OC)=C(c2ccccc2)C1=O	Betulinan A
CNP0204275.0	Cc1cnc(C(=O)CCO)c2[nH]c3ccccc3c12	Oxopropaline G
CNP0204877.0	COc1cc2oc(-c3ccccc3)cc(=O)c2c(O)c1OC	Mosloflavone
CNP0205018.0	c1cc2ccc3cccc4[nH]c(c1)c2c34	4H-Benzo[def]carbazole
CNP0205336.0	O=C1OCc2c1c(-c1ccc3c(c1)OCO3)c1cc3c(cc1c2O)OCO3	Taiwanin E
CNP0205360.0	C=Cc1cc(O)cc2ccc3c(C)c(O)ccc3c12	Dehydroeffusol
CNP0205792.0	CC1(C)C=Cc2c(O)cc(-c3cc4ccc(O)cc4o3)cc2O1	Moracin D
CNP0206776.0	COc1c(O)c(=O)n2c3ccccc3c3ccnc1c32	Nigakinone
CNP0207109.0	O=C1c2ccccc2C(=O)c2occc21	Naphtho[2,3-b]furan-4,9-dione

CNP0207402.0	COc1cc2c3c(ccc4cc(O)c(OC)c(c43)CO2)c1	Agrostophyllin
CNP0208142.0	O=C1C(O)=Cc2cccc3cc(-c4ccc(O)cc4)cc1c23	2-Hydroxy-8-(4-hydroxyphenyl)-1H-phenalen-1-one
CNP0210144.0	O=C1c2ccccc2C(=O)c2cc(O)ccc21	2-HYDROXYANTHRAQUINONE
CNP0210809.2	c1cc2cc3c(cc2o1)OC[C@H]1c2cc4c(cc2O[C@@H]31)OCO4	Neodulin
CNP0211413.0	O=C(c1ncc(-c2c[nH]c3ccccc23)[nH]1)c1c[nH]c2ccccc12	Deoxytopsentin
CNP0211540.0	COc1ccc2c(c1)C(=O)c1c3c(cc4ccnc-2c14)OCO3	Bianfugedine
CNP0213067.0	CC1(C)C=Cc2cc3c4c(oc3cc2O1)-c1ccc(O)cc1OC4	Anhydrotuberosin
CNP0214252.0	COc1c(-c2ccc3c(c2)OCO3)oc2c(ccc3occc32)c1=O	Pongapin
CNP0214831.0	Cc1ccc2c(c1)C(=O)c1cc(O)ccc1C2=O	7-Hydroxy-2-methylanthraquinone
CNP0215267.0	COc1cc2c(c(OC)c1)-c1ccc(O)cc1CC2	Orchinol
CNP0216540.0	C=Cc1nccc2c1[nH]c1c(O)cccc12	1-Vhb-carboline
CNP0216699.0	O=c1cc(-c2ccccc2)oc2ccccc12	FLAVONE
CNP0216887.0	Cc1coc2c1C(=O)c1ccc3c(C)cccc3c1C2=O	Isotanshinone I
CNP0216972.1	COc1cccc2c1C(=O)c1ccc3c(c1C2=O)C(=O)[C@@H](O)[C@@H](C)[C@H]3O	Rubiginone D2
CNP0217518.0	COc1cnc(C=O)c2[nH]c3ccccc3c12	Kumujancine
CNP0217740.0	O=C1OC(c2nccc3cc4c(cc23)OCO4)c2ccc3c(c21)OCO3	Hypecoumine
CNP0218940.0	COC(=O)c1cc(OC)c2[nH]c3ccccc3c2c1	Mukonine
CNP0219142.0	O=c1cc(-c2ccccc2)oc2c1ccc1occc12	LANCEOLATIN B
CNP0219609.0	Cc1cc2occ(C)c2cc2c(C=O)ccc1-2	azuleno[6,5-b]furan-5-carboxaldehyde, 3,8-dimethyl-
CNP0219704.0	COc1cc2c(=O)c3ccccc3oc2c(OC)c1OC	2,3,4-Trimethoxyxanthone
CNP0220929.0	COc1cc(OC)c2c(=O)cc(-c3ccc(OC)c(O)c3)oc2c1	3'-hydroxy-5,7,4'-trimethoxyflavone
CNP0223371.0	O=c1c2ccccc2oc2c(O)cccc12	4-Hydroxyxanthone

CNP0223554.0	COc1ccc(-c2cc(=O)c3ccccc3o2)cc1OC	3',4'-Dimethoxyflavone
CNP0224729.0	COc1cccc(-c2cc(=O)c3c(OC)cccc3o2)c1	5,3'-DIMETHOXYFLAVONE
CNP0225102.0	CC1(C)C=Cc2ccc3ccccc3c2O1	2H-Naphtho[1,2-b]pyran, 2,2-dimethyl-
CNP0225107.0	c1ccc2cc3c(cc2c1)[nH]c1ccccc13	5H-Benzo[b]carbazole
CNP0225405.0	COc1c(O)cc2c3c(cc4ccccc4c13)N(OC)C2=O	Piperlactam S
CNP0225695.0	COc1c(O)ccc2c1cnc1c3cc4c(cc3ccc21)OCO4	Decarine
CNP0226528.0	COc1cc(OC)c2c(ccc3cccc(O)c32)c1	Dehydroloroglossol
CNP0226881.0	COc1cc2oc(=O)cc(-c3ccccc3)c2cc1OC	6,7-Dimethoxy-4-phenylcoumarin
CNP0227071.0	COc1c(O)ccc2oc3ccccc3c(=O)c12	2-Hydroxy-1-methoxyxanthone
CNP0227072.0	CC(C)=CCC1=CC(=O)c2ccccc2C1=O	Deoxylapachol
CNP0229697.0	CC(C)=CCc1c(C=O)cc(O)c2[nH]c3ccccc3c12	Clausine D
CNP0230781.0	COc1cc(O)c2c(=O)cc(-c3ccccc3)oc2c1O	Isowogonin
CNP0231927.0	COc1cc2c(oc3c(OC)c(O)ccc32)c(OC)c1OC	beta-Cotonefuran
CNP0232623.0	COc1cc(O)c2c(=O)c3cccc(OC)c3oc2c1OC	1-hydroxy-3,4,5-trimethoxyxanthone
CNP0233375.0	CC1(C)C=Cc2c(ccc(-c3cc4ccc(O)cc4o3)c2O)O1	Glyinflanin H
CNP0233468.0	COc1cc2c(cc1OC)-c1c3c(cc4ccnc(c14)C2=O)OCO3	Dicentrinone
CNP0233560.0	COc1cc(C)c2c(=O)c(-c3ccccc3)coc2c1	5-Methyl-7-methoxyisoflavone
CNP0233688.0	COc1cc2[nH]c3ccccc3c(=O)c2c(O)c1OC	Xanthoxoline
CNP0234273.0	COc1ccc2c(=O)c(-c3ccccc3)coc2c1	7-methoxyisoflavone
CNP0234373.0	C=Cc1ncc(OC)c2c1[nH]c1ccccc12	Dehydrocrenatine
CNP0234402.0	CC1(C)C=CC2=C(O1)C(=O)c1ccccc1C2=O	Xyloidone
CNP0235326.1	C/C=C/c1ccc2c(c1)[C@@H](C)[C@H](c1ccc(O)cc1)O2	Conocarpan

CNP0235326.2	C/C=C/c1ccc2c(c1)[C@H](C)[C@@H](c1ccc(O)cc1)O2	(+)-Conocarpan
CNP0235404.0	CC(C)=CCc1c(O)c(CC=C(C)C)c2[nH]c3c(O)cccc3c(=O)c2c1O	Atalaphylline
CNP0235699.0	O=c1cc(-c2ccccc2)oc2c(O)ccc(O)c12	Primetin
CNP0235804.0	COc1cc(O)cc(-c2cc3c(OC)c(O)ccc3o2)c1	Gnetofuran B
CNP0236711.0	COC1=CC(=O)c2cc(-c3ccccc3)c3cc(O)c(OC)cc3c2C1=O	latinone
CNP0236913.0	COc1c(-c2ccccc2)oc2cccc(OC)c2c1=O	3,5-dimethoxyflavone
CNP0237465.0	O=C1C(O)=Cc2cccc3ccc(-c4ccccc4)c1c23	Anigorufone
CNP0238309.0	COc1cc2oc(-c3ccccc3)cc(=O)c2c(O)c1C	5-Hydroxy-7-methoxy-6-methylflavone
CNP0239041.0	COc1cc2ccnc3c2c(c1OC)-c1cc2c(cc1C3=O)OCO2	Oxonantenine
CNP0239760.0	CCc1nccc2c1[nH]c1ccccc12	1-ethyl-9H-pyrido[3,4-b]indole
CNP0239903.0	COc1cccc(-c2cc(=O)c3ccccc3o2)c1	3'-Methoxyflavone
CNP0241061.0	COc1cccc(OC)c1-c1cc(=O)c2c(O)c(OC)ccc2o1	Zapotinin
CNP0242072.0	COc1ccc2c(ccc3cc(OC)c(OC)c(OC)c32)c1	2,3,4,7-tetramethoxyphenanthrene
CNP0243115.0	Cc1cc2cccc3ccc4cccc1c4c32	4-METHYLPYRENE
CNP0244558.0	COc1cc2oc(-c3ccccc3)c(OC)c(=O)c2c(O)c1OC	Alnustin
CNP0246444.0	O=c1oc2ccccc2c2oc3ccccc3c(=O)c12	Frutinone A
CNP0246961.0	O=c1c(-c2ccc3c(c2)OCO3)coc2c3c(ccc12)OCO3	Maxima isoflavone A
CNP0247945.0	CC(C)=CCc1cc(-c2cc(=O)c3ccc(O)cc3o2)ccc1O	Kanzonol D
CNP0248185.1	COc1cccc2oc3c4c(cc(OC)c3c(=O)c12)O[C@H]1OC=C[C@@H]41	O-Methylsterigmatocystin
CNP0248320.0	O=C1C(O)=Cc2cccc3cccc1c23	2-hydroxy-1H-phenalen-1-one
CNP0248536.0	COC(=O)c1cc(O)c2[nH]c3ccccc3c2c1	Clausine E
CNP0249025.0	C=CC(C)(C)c1c2c(c(OC)c3ccc(=O)oc13)C=CC(C)(C)O2	Dentatin

CNP0249672.0	COc1cc2oc(-c3ccccc3)c(C)c2cc1O	Parvifuran
CNP0250210.0	CC(C)(O)c1cc2c(ccc3ccc(=O)oc32)o1	Oroselol
CNP0250715.0	COc1ccc2[nH]c3c(O)cc(C=O)cc3c2c1	Clausine I
CNP0251867.0	COc1ccccc1-c1cc(=O)c2c(O)cc(OC)c(OC)c2o1	Andrographin
CNP0251958.0	COc1ccccc1-c1coc2cc3c(c(O)c2c1=O)OCO3	Irisone A
CNP0252776.0	COc1cc(-c2ccc3cccc4c3c2C(=O)C(O)=C4)ccc1O	Musanolone F
CNP0253191.0	COc1cc2c(-c3ccc4c(c3)OCO4)c3c(cc2cc1O)COC3=O	Daurinol
CNP0253591.0	COc1cc2c(c(OC)c1C)C(=O)c1ccccc1C2=O	Rubiadin dimethyl ether
CNP0254320.0	COc1cccc2oc3ccc(O)c(OC)c3c(=O)c12	2-Hydroxy-1,8-dimethoxyxanthone
CNP0255182.0	CC1=CC(=O)c2[nH]c3ccccc3c2C1=O	Murrayaquinone A
CNP0255210.0	COc1c(-c2ccccc2)oc2c(ccc3occc32)c1=O	Karanjin
CNP0255606.0	O=C(Cc1ccccc1)c1nccc2c1[nH]c1ccccc12	Eudistomin T
CNP0256026.0	O=Cc1cc2c(cc1O)[nH]c1ccccc12	Mukonal
CNP0258893.0	COc1ccc(-c2coc3cc4c(c(OC)c3c2=O)OCO4)cc1	Irisolone methyl ether
CNP0259836.0	COc1c(OC)c2nccc3c4ccccc4n(c1=O)c23	4,5-DIMETHOXYCANTHIN-6-ONE
CNP0259934.0	COc1cc(-c2coc3cc4c(c(OC)c3c2=O)OCO4)cc(OC)c1OC	Irisflorentin
CNP0260625.0	O=c1c2ccccc2oc2ccc(O)cc12	2-Hydroxyxanthone
CNP0260729.0	Brc1ccc2[nH]c3c(C4=NCCC4)nccc3c2c1	Eudistomin H
CNP0260963.0	COc1ccc2[nH]c3c(C=O)c(C)c(OC)c(O)c3c2c1	Carbazomycin F
CNP0261153.0	COc1ccc(-c2cc3cc(OC)ccc3o2)cc1	5-methoxy-2-(4-methoxyphenyl)benzofuran
CNP0261262.0	O=c1c2ccccc2oc2cc3c(cc12)OCO3	2,3-methylenedioxyxanthone
CNP0261785.0	O=c1cc(-c2ccccc2)oc2cc3c(c(O)c12)OCO3	Cochliophilin A

CNP0262134.0	COc1c2ccoc2cc2oc(-c3ccccc3)cc(=O)c12	Pinnatin
CNP0262352.0	N=c1ccc2c(-c3ccccc3C(=O)O)c3ccc(N)cc3oc-2c1	Rhodamine 110
CNP0263116.0	O=c1cc(C=Cc2ccccc2)oc2cccc(O)c12	5-hydroxy-2-styrylchromone
CNP0263220.0	CC(C)=CCc1cc(-c2cc3cc4c(cc3o2)OCO4)c(O)cc1O	2-(2,4-dihydroxy-5-prenylphenyl)-5,6-methylenedioxybenzofuran
CNP0263819.0	O=C1C(=O)c2ccccc2-c2ccccc21	Phenanthrene-9,10-dione
CNP0265814.0	COc1c(OC)c2c3c(nccc3c1OC)-c1cc(O)ccc1-2	norruffscine
CNP0266880.0	COc1cc(OC)c2nc3occc3c(OC)c2c1	Maculosidine
CNP0269087.0	CC(C)=CCc1c(O)c(C=O)cc2c1[nH]c1cc(O)ccc12	7-Hydroxyheptaphylline
CNP0269097.0	CC1(C)C=Cc2c(c(C=O)cc3c2[nH]c2ccccc23)O1	Murrayacine
CNP0269307.0	COc1ccc2c(=O)c(-c3ccc(O)c(OC)c3)coc2c1	Sayanedine
CNP0270121.0	COc1cc(OC)c2c(c1)oc1c3ccc(O)cc3oc(=O)c12	Wairol
CNP0274606.0	CC1=CCc2c(ccc3cc(-c4cc(O)cc(O)c4)oc23)OC1	Moracin G
CNP0276307.0	CC1=COC2=C(C)C(=O)C(=O)c3c(C)ccc1c32	Mansonone F
CNP0276388.0	O=c1oc2ccccc2c2oc3ccccc3c12	Coumestan
CNP0277146.0	Cn1cc2ccnc3c2c1C(=O)C(N)=C3Cl	Isobatzelline E
CNP0279735.0	O=C1OCc2c1cc1ccc3c(c1c2-c1ccc2c(c1)OCO2)OCO3	Helioxanthin
CNP0280443.0	O=C1c2[nH]c3ccccc3c2C(=O)c2cnc3ccccc3c21	CALOTHRIXIN B
CNP0280497.0	CC1(C)C=Cc2ccc3c(=O)c4c(O)cccc4oc3c2O1	Dehydrocycloguanandin
CNP0281512.0	C=Cc1c(C)c(O)cc2c1-c1ccc(O)c(C)c1CC2	Juncusol
CNP0281835.0	COc1cc2c(cc1-c1cc3ccc(O)cc3o1)OCO2	Cicerfuran
CNP0282699.0	COc1cccc2c1C(=O)c1c(OC)cc(C)cc1C2=O	1,8-dimethoxy-3-methylanthracene-9,10-dione
CNP0283010.0	COc1cc(OC)c2c(=O)cc(-c3ccccc3)oc2c1	5,7-Dimethoxyflavone

CNP0283796.0	Cc1ccc2c(c1O)C(=O)c1ccccc1C2=O	1-Hydroxy-2-methylanthraquinone
CNP0284187.0	COc1cccc2oc3c(O)cccc3c(=O)c12	5-Hydroxy-1-methoxyxanthone
CNP0284605.0	COc1cc2c(OC)c3c(c(-c4ccc5c(c4)OCO5)c2cc1OC)C(=O)OC3	Justicidin A
CNP0285640.0	COc1cc2c(cc1O)C(c1ccccc1)=CCO2	Dalbergichromene
CNP0286528.0	COc1ccc2c(c1)C(=O)c1nccc3cc4c(c-2c13)OCO4	Lanuginosine
CNP0287309.0	COc1cc(O)c2c(=O)c(-c3ccccc3)coc2c1	5-Hydroxy-7-methoxyisoflavone
CNP0288378.0	O=C1C=C(Nc2ccccc2)C(=O)c2ccccc21	2-Anilino-1,4-naphthoquinone
CNP0289498.0	CC1(C)C=Cc2c(c3ccccc3[nH]c2=O)O1	Flindersine
CNP0290384.0	Cc1c2cnccc2c(C=O)c2[nH]c3ccccc3c12	13-Oxoellipticine
CNP0290567.0	COc1c2ccoc2nc2c(OC)c3c(cc12)OCO3	Flindersiamine
CNP0292729.0	COc1cccc(C2=CC(=O)C=CC2=O)c1	2-(3-methoxyphenyl)cyclohexa-2,5-diene-1,4-dione
CNP0292914.0	COc1c(-c2ccccc2)oc2c3c(ccc2c1=O)OC(C)(C)C=C3	Karanjachromene
CNP0293814.0	COC(=O)c1cc2c(cc1OC)[nH]c1ccccc12	Clausine L
CNP0295830.0	O=c1cc(-c2ccccc2)oc2c1ccc1ccccc12	alpha-Naphthoflavone
CNP0296580.0	C=Cc1cc(OC)c2c(c1)c(=O)oc1c3cccc(O)c3c(OC)cc12	Defucogilvocarcin V
CNP0297762.0	COc1c(OC)c(OC)c2c(=O)c(OC)c(-c3ccccc3)oc2c1OC	3,5,6,7,8-Pentamethoxyflavone
CNP0298366.0	Cc1coc2c1C(=O)C(=O)c1ccc3c(C)cccc3c1-2	Isotanshinone II
CNP0300085.0	COc1cc2oc3ccccc3c(=O)c2cc1OC	2,3-dimethoxyxanthen-9-one
CNP0301048.0	O=c1c2ccccc2[nH]c2cccc(O)c12	1-hydroxyacridone
CNP0301680.0	CC(C)=CCOc1cc2ccc(=O)oc2c2ccoc12	HERATOMIN
CNP0303902.0	O=c1c(-c2ccc3c(c2)OCO3)coc2cc(O)ccc12	Pseudobaptigenin
CNP0303982.0	CC(C)=CCc1cccc2nc3c(C(=O)O)cccc3nc12	6-(3-Methyl-2-butenyl)-1-phenazinecarboxylic acid

CNP0304443.0	O=c1oc2cc(O)ccc2c2oc3cc4c(cc3c12)OCO4	Medicagol
CNP0304805.0	COc1c(OC)c(O)c2c(=O)c3cccc(OC)c3oc2c1OC	1-Hydroxy-2,3,4,5-tetramethoxyxanthone
CNP0304915.0	CC(=O)c1cc2c(o1)C(=O)c1ccccc1C2=O	Napabucasin
CNP0305018.0	c1ccc2c(c1)ccc1c3ccccc3oc21	Benzo[b]naphtho[2,1-d]furan
CNP0305447.0	Oc1ccc2[nH]c3c(-c4ccc[nH]4)nccc3c2c1	Eudistomin M
CNP0306397.0	CC(C)=CCc1c(O)c(C=O)cc2c1[nH]c1ccccc12	Heptaphylline
CNP0306686.0	COc1cc(OC)c2c(=O)c(O)c(-c3ccccc3)oc2c1	5,7-DIMETHOXY-3-HYDROXYFLAVONE
CNP0307270.0	Cc1nccc2c1[nH]c1ccc(Br)cc12	6-bromo-1-methyl-9H-pyrido[3,4-b]indole
CNP0307672.0	COc1cccc(-c2cc(=O)c3c(OC)c(OC)ccc3o2)c1	3',5,6-Trimethoxyflavone
CNP0308030.0	COc1cc(O)cc(-c2cc3cc(O)c(OC)cc3o2)c1	Moracin B
CNP0308988.0	Cn1c2ccccc2c(=O)c2ccoc21	Isodictamnine
CNP0309366.0	COc1ccc2ccc3ccc(OC)c(O)c3c2c1	Thebaol
CNP0309779.0	Cc1c(O)cc2ccc3c(C)c(O)cc4ccc1c2c43	2,7-Dihydroxy-1,6-dimethylpyrene
CNP0309948.0	c1ccc2c(c1)ccc1c3ccccc3[nH]c21	11H-Benzo[a]carbazole
CNP0310550.0	COc1ccc2oc(-c3ccccc3)c(O)c(=O)c2c1	3-Hydroxy-6-methoxyflavone
CNP0310868.0	Brc1ccc2c(Br)c(Br)[nH]c2c1	2,3,6-tribromo-1H-indole
CNP0311398.0	COc1cc(-c2ccccc2)c(O)c(O)c1-c1ccccc1	Terferol
CNP0311823.0	COc1ccc2c(OC)c3ccoc3nc2c1	Evolitrine
CNP0311835.0	COc1c(C)c(O)c2c(=O)cc(-c3ccccc3)oc2c1C	Desmosflavone
CNP0312219.0	C=Cc1cc(O)cc2c1-c1ccc(O)c(C)c1CC2	EFFUSOL
CNP0312750.0	COc1cccc2nc3occc3c(OC)c12	4,5-DIMETHOXYFURO[2,3-B]QUINOLINE
CNP0313414.0	COc1cc2oc3c(O)cccc3c(=O)c2c(O)c1CC=C(C)C	CUDRAXANTHONE

CNP0314439.0	COc1ccc(-c2cc(=O)c3ccccc3o2)cc1	4'-Methoxyflavone
CNP0314585.0	COc1cc2[nH]c3ccccc3c2cc1C=O	glycosinine
CNP0314618.0	O=C1c2ccccc2C(=O)c2c(O)cccc21	1-HYDROXYANTHRAQUINONE
CNP0314825.0	COc1cc2c(OC)cnc3c2c(c1OC)-c1ccccc1C3=O	Splendidine
CNP0314850.0	COc1cc2oc(-c3ccccc3)cc(=O)c2c(OC)c1OC	5,6,7-Trimethoxyflavone
CNP0315189.0	COc1ccc(-c2coc3c4c(ccc3c2=O)OCO4)cc1	Maximaisoflavone H
CNP0316994.0	COc1ccc(-c2coc3cc(O)c(OC)cc3c2=O)cc1	Afrormosin
CNP0318031.0	C=C(C)c1cc2cc(OC)c(OC)cc2o1	5,6-dimethoxy-2-isopropenylbenzofuran
CNP0318062.0	CC(C)=CCc1cccc2nc3cccc(C(=O)O)c3nc12	Endophenazine A
CNP0318221.0	COc1ccc2c(=O)c(OC)c(-c3ccccc3)oc2c1	3,7-Dimethoxyflavone
CNP0318807.0	COc1ccc(-c2ccc3cccc4c3c2C=C(O)C4=O)cc1	4'-O-Methylirenolone
CNP0319079.0	COc1ccc2oc(-c3ccccc3)cc(=O)c2c1OC	5,6-Dimethoxyflavone
CNP0320480.0	Brc1ccc2c(c1)[nH]c1c(C3=NCCC3)nccc12	Eudistomin G
CNP0320511.0	COc1c(OC)c(OC)c2c(c1O)c(=O)c1ccccc1n2C	Normelicopicine
CNP0320714.0	COc1cc2c(=O)c(-c3ccc4c(c3)OCO4)coc2cc1O	fujikinetin
CNP0321173.0	COc1ccc2c(=O)cc(-c3ccccc3)oc2c1OC	7,8-Dimethoxyflavone
CNP0321376.0	O=C1c2ccccc2-c2c3c(cc4ccnc1c24)OCO3	Liriodenine
CNP0322591.0	COc1c(O)cc2c3c(cc4ccccc4c13)NC2=O	Aristolactam AII
CNP0323192.0	COc1cc2nc3occc3c(OC)c2cc1OC	Kokusaginine
CNP0323763.0	C=Cc1ncc(OC)c2c1[nH]c1c(OC)cccc12	Dehydrocrenatidine
CNP0323833.0	COc1ccc2oc(-c3cccc(OC)c3OC)cc(=O)c2c1	6,2',3'-Trimethoxyflavone
CNP0324429.0	c1ccc2c(c1)ccc1[nH]c3ccccc3c12	7H-Benzo[c]carbazole

CNP0325033.0	COc1cc2c(=O)c3ccccc3oc2c(O)c1OC	4-hydroxy-2,3-dimethoxyxanthone
CNP0325037.0	COc1ccc(-c2coc3cccc(O)c3c2=O)cc1	Pallidiflorin
CNP0326183.0	COc1cccc2c1[nH]c1cncc(OC)c12	4,8-Dimethoxy-9H-pyrido[3,4-b]indole
CNP0327689.0	C=Cc1nccc2c1[nH]c1ccccc12	1-ethenyl-9h-pyrido[3,4-b]indole
CNP0327812.0	Oc1ccc2ccc3cccc4ccc1c2c34	1-HYDROXYPYRENE
CNP0327879.0	COc1c2c(cc3oc(-c4ccccc4)cc(=O)c13)OCO2	5-Methoxy-6,7-methylenedioxyflavone
CNP0329088.0	CC(C)=CCc1c(O)ccc2c(=O)c(-c3ccc(O)cc3)coc12	8-Prenyldaidzein
CNP0329181.0	O=Cc1ccc2c(c1)C(=O)c1ccccc1C2=O	9,10-dioxo-9,10-dihydroanthracene-2-carbaldehyde
CNP0330726.0	COc1ccc2c3c([nH]c2c1)C(C)=NCC3	harmaline
CNP0331809.0	Cc1ccc2c(c1)C(=O)c1c(O)cccc1C2=O	Barleriaquinone I
CNP0332262.0	O=c1c(O)c(-c2ccccc2)oc2cccc(O)c12	3,5-DIHYDROXYFLAVONE
CNP0332453.0	Oc1ccc2c(c1)OCc1c-2oc2cc3c(cc12)OCO3	Dehydromaackiain
CNP0332640.0	COc1ccc(-c2cc(=O)c3c(OC)cccc3o2)cc1	5,4'-dimethoxyflavone
CNP0333341.0	COc1cc(OC)c2c(ccc3cc(OC)c(O)cc32)c1	Batatasin I
CNP0333434.0	COc1cc2c(cc1-c1cc3cc4ccoc4cc3oc1=O)OCO2	Pachyrrhizin
CNP0335320.0	COc1cc2oc3c(OC)cccc3c(=O)c2c(O)c1OC	1-Hydroxy-2,3,5-trimethoxyxanthone
CNP0336127.0	O=c1cc(-c2ccccc2O)oc2ccccc12	2'-Hydroxyflavone
CNP0338133.0	C1=C(c2ccccc2)c2ccccc2C1	3-Phenyl-1H-indene
CNP0338175.0	COc1ccc(-c2oc3ccccc3c(=O)c2OC)cc1	3,4'-dimethoxyflavone
CNP0338803.0	O=c1c(-c2ccccc2)c1-c1ccccc1	Diphenylcyclopropenone
CNP0345294.0	Cc1cc2occ(C)c2cc2c(C)ccc1-2	Linderazulene
CNP0349903.0	Nc1ccc(O)c2c1C(=O)c1ccccc1C2=O	1-AMINO-4-HYDROXYANTHRAQUINONE

CNP0349972.0	COc1cccc(-c2cc(=O)c3ccc(OC)cc3o2)c1	7,3'-dimethoxyflavone
CNP0350107.0	Cc1cc2c(cc1C)C(=O)c1ccccc1C2=O	2,3-Dimethylanthraquinone
CNP0350147.0	Nc1cccc2c1C(=O)c1ccccc1C2=O	1-AMINOANTHRAQUINONE
CNP0355815.0	CC(C)=CCc1c2ccoc2c(O)c2oc(=O)ccc12	Alloimperatorin
CNP0356121.0	COc1ccc2c3c([nH]c2c1)C(=O)C=C(C)C3=O	Koeniginequinone A
CNP0357048.0	c1ccc2c(c1)-c1cccc3ccnc-2c13	1-Azafluoranthene
CNP0358144.0	COc1c(O)c2nccc3c4ccccc4n(c1=O)c23	Picrasidine Q
CNP0358610.0	O=C1C=Cc2cc3ccccc3cc2C1=O	1,2-Anthraquinone
CNP0358965.0	COc1ccc2c(c1)oc(=O)c1c3ccc(OC)cc3oc21	Coumestrol dimethyl ether
CNP0360635.0	COC1=C(c2ccccc2)C(=O)C2=C(C1=O)c1ccccc1CO2	Betulinan B
CNP0360832.0	O=c1ccc2nccc3c4ccccc4n1c23	Canthin-6-one
CNP0360853.0	COC(=O)c1ccccc1-c1c2ccc(=N)cc-2oc2cc(N)ccc12	Rhodamine 123 free base
CNP0361125.0	O=c1cc(-c2ccccc2)c2ccc(O)cc2o1	7-Hydroxy-4-phenylcoumarin
CNP0363551.0	COC(=O)c1cc(O)c2[nH]c3ccccc3c2c1CC=C(C)C	Clausine F
CNP0365088.0	O=c1c2ccccc2[nH]c2cc3c(=O)c4ccccc4[nH]c3cc12	Quinacridone
CNP0366241.2	Oc1ccc2c(c1)OC[C@@H]1c3cc4c(cc3O[C@H]21)OCO4	(+)-Maackiain
CNP0369127.0	COc1c(Br)cc2c(Br)c[nH]c2c1Br	3,5,7-tribromo-6-methoxy-1H-indole
CNP0374158.0	O=C1OCc2c1cc1ccc3c(c1c2-c1cc(O)c2c(c1)OCO2)OCO3	Justicinol
CNP0376927.0	O=C1OCc2cc3cc4c(cc3c(-c3ccc5c(c3)OCO5)c21)OCO4	Taiwanin C
CNP0377194.0	c1ccc2c(c1)Oc1cccc3cccc-2c13	Benzo[kl]xanthene
CNP0378247.0	Nc1ccc2ncnc3c2c1C(=O)c1ccccc1-3	6-amino-7H-benzo[e]perimidin-7-one
CNP0380943.0	COc1cc(OC)c2c(=O)c3cccc(OC)c3oc2c1	1,3,5-trimethoxyxanthone

CNP0382870.0	COc1cc(O)c2c(=O)c3cc(OC)ccc3oc2c1	1-Hydroxy-3,7-dimethoxyxanthone
CNP0385863.0	Cn1c2ccccc2c(=O)c2c(O)cc3c(c21)C=CC(C)(C)O3	Noracronycine
CNP0389659.0	COc1c(O)c(C)cc2c1C(=O)c1ccccc1C2=O	Digitolutein
CNP0392709.0	COc1cc2nc3cc(CO)ccc3oc-2cc1=O	4-Demethoxymichigazone
CNP0395155.0	COc1ccccc1-c1cc(=O)c2cccc(OC)c2o1	8,2'-DIMETHOXYFLAVONE
CNP0396733.0	O=C1C=C(Nc2ccccc2)c2ccccc2C1=O	4-(Phenylamino)naphthalene-1,2-dione
CNP0402074.0	COc1ccc2nc3occc3c(OC)c2c1	Pteleine
CNP0404535.0	COc1cc2c(cc1O)C(=O)c1nccc3cc(OC)c(OC)c-2c13	Atheroline
CNP0405375.0	ON=c1c2ccccc2oc2ccccc12	Xanthone oxime
CNP0405810.0	COc1c(OC)c2c(OC)c3ccoc3cc2oc1=O	Halfordin
CNP0408855.0	CCc1ncc(OC)c2c1[nH]c1c(O)cccc12	Picrasidine J
CNP0409718.0	Cc1[nH]ccc2c1[nH]c1cc(=O)ccc12	Harmol
CNP0410358.0	COc1c2ccoc2c(OCC=C(C)C)c2oc(=O)ccc12	Phellopterin
CNP0416205.0	COC1=Cc2cc(OC)c(O)c3ccc(-c4ccccc4)c(c23)C1=O	xiphidone
CNP0419453.0	Cc1c2ccncc2c(C)c2c1[nH]c1ccccc12	ellipticine
CNP0420155.0	COc1ccc(-c2cc(=O)c3ccc(O)cc3o2)cc1	Pratol
CNP0421927.0	COc1ccc2c(=O)c3ccccc3oc2c1	3-Methoxyxanthone
CNP0437245.0	O=C1C(=O)c2cccc3cccc1c23	Acenaphthenequinone
CNP0445310.0	O=c1ccc2c3ccc(=O)c4cccc(c5cccc1c52)c43	3,10-Perylenedione
CNP0454546.0	C1=Cc2ccccc2Nc2ccccc21	Iminostilbene
CNP0458543.0	O=C1C=Cc2ccc3ccccc3c2C1=O	3,4-Phenanthrenedione
CNP0472610.0	O=c1ccc2ccccc2c2ccccc12	5H-Dibenzo[a,c]cyclohepten-5-one

CNP0474883.0	O=Cc1coc2c(Br)cc(Br)cc2c1=O	6,8-Dibromo-3-formylchromone
CNP0483233.0	O=Cc1c(-c2ccccc2)[nH]c2ccccc12	2-Phenyl-1H-indole-3-carbaldehyde
CNP0483240.0	CC[n+]1c(-c2ccccc2)c2cc(N)ccc2c2ccc(N)cc21.[Br-]	ETHIDIUM BROMIDE
CNP0492013.0	O=c1c2ccccc2[nH]c2ccccc12	acridone
CNP0496836.0	c1ccc(-c2ccc3ccccc3[o+]2)cc1	Flavylium
CNP0502051.0	O=P(O)(O)C(O)(Cc1cccc(-c2cccc3c2oc2ccccc23)c1)P(=O)(O)O	BPH-629
CNP0502715.0	COc1cc(O)c2c(=O)c3ccccc3oc2c1	Xanthen-9-one, 1-hydroxy-3-methoxy-
CNP0506196.0	O=c1ccc2c3c(cc[n+]2[O-])c2ccccc2n13	Canthin-6-one N-oxide
CNP0506320.0	O=C1c2ccccc2C(=O)c2ncccc21	benzo[g]quinoline-5,10-dione
CNP0511918.0	Cc1ncc2c(c1O)COC2c1ccc(Cl)cc1	Cicletanine
CNP0517817.0	COc1cc2cc[n+](C)c3c2c(c1O)-c1cc2c(cc1C3=O)OCO2	Nandazurine
CNP0526269.0	[Cl-].c1ccc(-c2ccc3ccccc3[o+]2)cc1	2-Phenyl-1-benzopyrylium chloride
CNP0526765.0	O=C1CC=Cc2cc3ccccc3cc21	Anthracenone
CNP0530584.0	C[n+]1c2cc(N)ccc2cc2ccc(N)cc21	3,6-Diamino-10-methylacridinium
CNP0539127.0	Cc1ncc(C)c2c1[nH]c1ccccc12	1,4-dimethyl-9H-pyrido[3,4-b]indole
CNP0552302.0	O=Cc1c2ccccc2nc2ccccc12	Acridine-9-carbaldehyde
CNP0559972.0	C1=CC(c2ccccc2)Oc2ccccc21	2-phenyl-2H-chromene
CNP0561658.0	C1=Cc2c(ccc3ccccc23)CC1	1,2-Dihydrophenanthrene
CNP0568671.0	Cn1c2ccccc2c2cc[n+](C)cc21	2,9-dimethyl-beta-carbolinium
CNP0568820.0	O=C1NC(=O)c2c1c1c3ccc(O)cc3[nH]c1c1[nH]c3cc(O)ccc3c21	arcyriaflavin C
CNP0573878.0	CC[n+]1c(-c2ccccc2)c2cc(N)ccc2c2ccc(N)cc21	Ethidium
CNP0575419.0	Cc1cc(O)c2c(c1)OC(C)(C)c1ccc(C)cc1-2	Cannabiorcol

CNP0575 490.0	c1cc2c3c(c1)c1cc4c(cc1c[n+]3CC2)OCO4	Anhydrolycorinium
CNP0577 327.0	O=C(O)c1cc2c(c3c1c([N+](=O)[O-])cc1ccccc13)OCO2	Aristolochic acid B
CNP0579 162.0	COc1cc2c(c(OC)c1CO)C(=O)c1ccccc1C2=O	2-(Hydroxymethyl)-1,3-dimethoxyanthraquinone
CNP0579 989.0	CC1c2ccccc2-c2ccccc21	9-Methylfluorene
CNP0584 117.0	O=C1N=c2ccccc2=C1c1c(O)[nH]c2ccccc12	Isoindigotin
CNP0586 952.0	O=C1NC(=O)c2c1c1nc[nH]c1c1[nH]c3ccccc3c21	GRANULATIMIDE
CNP0589 461.0	O=c1cc(C=Cc2ccccc2)oc2ccccc12	styrylchromone
CNP0589 474.0	Nc1cccc2c1-c1ccccc1C2=O	4-Amino-9-fluorenone
CNP0595 330.0	O=C1c2ccccc2-[n+]2ccc3c([nH]c4ccccc43)c21.[Cl-]	Fascaplysin
CNP0596 265.0	O=C(O)c1cc2ccc3cccc4ccc(c1)c2c34	Pyrene-2-carboxylic acid
CNP0598 085.0	Cc1nccc2c1[nH]c1cc(O[11CH3])ccc12	Harmine C-11
CNP0603 222.0	O=C1c2ccccc2-[n+]2ccc3c([nH]c4ccccc43)c21	Fascaplisine
CNP0604 632.0	COC(=O)c1cc2c(c3c1c([N+](=O)[O-])cc1c(OC)cccc13)OCO2	Aristolochic acid I methyl ester
CNP0240 311.1	COc1cc(-c2ccc3cccc4c3c2C(=O)[C@H](O)[C@H]4O)ccc1O	DTXSID201128077
CNP0219 228.0	CC1(C)C=Cc2c(cc(O)c3c(=O)c4cccc(O)c4[nH]c23)O1	atolaphyllidine
CNP0573 705.0	O=C1NC(=O)c2c1c1c3ccccc3[nH]c1c1[nH]c3c(Cl)cccc3c21	5-chloro-3,13,23-triazahexacyclo[14.7.0.02,10.04,9.011,15.017,22]tricosa-1,4(9),5,7,10,15,17,19,21-nonaene-12,14-dione
CNP0223 797.0	Oc1cc2c(cc1Br)[nH]c1c(-c3ccc[nH]3)nccc12	7-Bromo-1-(1H-pyrrol-2-yl)-9H-pyrido(3,4-b)indol-6-ol
CNP0323 387.0	COc1c2c(cc3oc(-c4ccc(O)cc4O)cc13)OC(C)(C)C=C2	Neoraufurane
CNP0161 932.0	C=CC(C)(C)c1cc(O)c2oc3c(ccc4ccoc43)c(=O)c2c1O	Dulciol D
CNP0262 280.0	COc1ccc(-c2cc3cc(O)c(OC)cc3o2)c(O)c1	Sainfuran
CNP0300	COc1cc2cc(-c3ccc(O)cc3O)oc2cc1OC	2-(2,4-Dihydroxyphenyl)-5,6-

862.0		dimethoxybenzofuran
CNP0306125.0	COc1c(O)cc(CC=Cc2ccccc2O)c(OC)c1OC	DTXSID60701602
CNP0311220.0	Oc1ccc(-c2cc3cc4c(cc3o2)OCO4)c(O)c1	2',4'-Dihydroxy-5,6-methylenedioxy-2-phenylbenzofuran
CNP0277462.0	CC1(C)C=Cc2c(O)ccc(-c3cc4ccc(O)cc4o3)c2O1	Glabrocoumarone A
CNP0215161.0	COc1cc(-c2ccccc2)c(OC)c(O)c1-c1ccc(O)cc1	4''-Deoxyterphenyllin
CNP0121466.0	O=c1c(O)cc2cc(O)c3oc4ccccc4c4ccc1c2c34	Lachnanthofluorene
CNP0237856.0	O=c1c(-c2ccc(Cl)cc2)coc2cc(O)cc(O)c12	3-(4-chlorophenyl)-5,7-dihydroxy-4H-chromen-4-one
CNP0283205.0	COc1ccc(-c2cc3ccc(O)cc3o2)c(O)c1	Centrolobofuran
CNP0555584.0	O=C1c2ccccc2C(=O)c2c(O)c(Cl)c(Cl)c(O)c21	2,3-dichloro-1,4-dihydroxyanthracene-9,10-dione
CNP0226932.0	Cc1c(O)cc2oc(-c3ccccc3)cc(=O)c2c1O	Strobochrysin
CNP0175577.0	O=c1cc(-c2cccc(O)c2O)oc2ccccc12	2',3'-Dihydroxyflavone
CNP0376877.0	Nc1ccc(-c2coc3cc(O)ccc3c2=O)cc1	3-(4-aminophenyl)-7-hydroxy-4H-chromen-4-one
CNP0189989.0	COC(=O)c1ncc(O)c2c1[nH]c1ccccc12	4-hydroxy-1-methoxycarbonyl-beta-carboline
CNP0328286.0	O=C1OC(=O)C(c2c[nH]c3ccccc23)=C1c1c[nH]c2ccccc12	Pityrianhydride
CNP0192622.0	Oc1cc(O)cc(-c2cc3ccccc3o2)c1	1,3-benzenediol, 5-(2-benzofuranyl)-
CNP0314163.0	COc1ccc2c(c1)[nH]c1cc(O)c(C=O)cc12	2-Hydroxy-7-methoxy-9H-carbazole-3-carbaldehyde
CNP0207880.0	COc1cc(C=O)cc2c1[nH]c1cc(O)ccc12	7-hydroxy-1-methoxy-9H-carbazole-3-carbaldehyde
CNP0247243.0	COc1ccc2c(c1)[nH]c1c(C(N)=O)nccc12	Harmic amide
CNP0529871.0	O=C1NC(=O)c2[nH]c3ccccc3c2C1=O	1H-Pyrido[3,4-b]indole-1,3 4(2H,9H)-trione
CNP0216925.0	Cc1c(O)cc2ccc3cc(O)cc4ccc1c2c34	1-Methylpyrene-2,7-diol
CNP0186649.0	Cc1c2ccncc2c(C)c2c1[nH]c1ccc(O)cc12	5,11-dimethyl-6H-pyrido[4,3-b]carbazol-9-ol
CNP0162260.0	C=Cc1c(O)c(C)cc2c1-c1ccc(O)c(C)c1CC2	2,6-Phenanthrenediol, 5-ethenyl-9,10-dihydro-1,7-dimethyl-

CNP0113773.0	C=Cc1cc(O)c(C)c2c1-c1ccc(O)c(C)c1CC2	1-Methyleffusol
CNP0434619.0	Oc1c(Br)cc2[nH]c3cnccc3c2c1Br	5,7-dibromo-9H-pyrido[3,4-b]indol-6-ol
CNP0328144.0	Cc1c2ccncc2c(CO)c2c1[nH]c1ccccc12	6H-Pyrido(4,3-b)carbazole-11-methanol, 5-methyl-
CNP0321137.0	COc1cc(C)cc2c1[nH]c1cc(O)ccc12	7-Hydroxy-1-methoxy-3-methylcarbazole
CNP0512285.0	O=C1Nc2ccccc2C1=Cc1ccc(O)cc1	3-(4-Hydroxybenzylidenyl)indolin-2-one
CNP0151836.0	O=Cc1cc(O)c2[nH]c3ccccc3c2c1	Demethylmurrayanine
CNP0129050.0	O=c1c2ccccc2[nH]c2ccc(O)cc12	2-Hydroxy-10H-acridin-9-one
CNP0321861.0	OCCc1nccc2c1[nH]c1ccccc12	2-(9H-beta-Carbolin-1-yl)ethanol
CNP0287687.0	NC(=O)c1nccc2c1[nH]c1ccccc12	9H-Pyrido[3,4-b]indole-1-carboxamide
CNP0143503.0	Cc1ccc2[nH]c3ccc(O)cc3c2c1	GLYCOZOLININE
CNP0187910.0	Cc1cc(O)c2[nH]c3ccccc3c2c1	3-Methyl-9H-carbazol-1-ol
CNP0267777.0	Cc1cc2c(cc1O)[nH]c1ccccc12	3-methyl-9H-carbazol-2-ol
CNP0310509.0	O=c1c2ccccc2[nH]c2c1[nH]c1ccccc12	NSC357573
CNP0426020.0	O=c1c2ccccc2[nH]c2[nH]c3ccccc3c12	5H,6H,11H-indolo[2,3-b]quinolin-11-one
CNP0334198.0	COc1ccc2c(c1O)OCc1c-2oc2c(OC)c(OC)ccc12	Bryacarpene 4
CNP0303723.0	COC(=O)c1c(-c2ccc(O)cc2OC)oc2cc(OC)ccc12	Methyl 2-(2-methoxy-4-hydroxyphenyl)-6-methoxy-3-benzofurancarboxylate
CNP0114107.0	COc1cc2oc3c4ccc(O)cc4oc(=O)c3c2cc1OC	3-Hydroxy-8,9-dimethoxycoumestan
CNP0133590.0	COc1c(O)c(OC)c2c(oc3c(OC)cccc32)c1OC	6-Methoxy-alpha-pyrufuran
CNP0254483.0	COc1cc(OC)c2c(-c3ccc(OC)c(O)c3)cc(=O)oc2c1	3'-Hydroxy-5,7,4'-trimethoxy-4-phenylcoumarin
CNP0102783.0	COc1ccc(-c2cc(=O)oc3c(O)c(OC)cc(OC)c23)cc1	Exostemin
CNP0040765.0	COc1ccccc1-c1oc2c(OC)c(OC)ccc2c(=O)c1O	3-Hydroxy-2',7,8-trimethoxyflavone
CNP0239	COc1cc(-c2cc(=O)c3ccc(O)cc3o2)cc(OC)c1OC	7-Hydroxy-2-(3,4,5-

749.0		trimethoxyphenyl)-4H-chromen-4-one
CNP0081233.0	COc1ccc(-c2cc(=O)c3c(O)cc(OC)cc3o2)c(OC)c1	Noratocarpetin 7,2',4'-trimethyl ether
CNP0259889.0	COc1ccc(-c2cc(=O)c3c(OC)cc(OC)c(O)c3o2)cc1	8-Hydroxy-4',5,7-trimethoxyflavone
CNP0299478.0	COc1c(OC)c(O)c2c(=O)cc(-c3ccccc3)oc2c1OC	Alnetin
CNP0239204.0	COc1cc2c(cc1O)C(=O)c1nccc3cc4c(c-2c13)OCO4	Machigline
CNP0316198.0	COc1cc(O)c2c(=O)c3ccc(OC)c(OC)c3oc2c1	9H-Xanthen-9-one, 1-hydroxy-3,5,6-trimethoxy-
CNP0383801.0	Cc1c(O)ccc2c(-c3cc4c(ccc5ccccc54)oc3=O)cc(=O)oc12	2-(7-hydroxy-8-methyl-2-oxo-2H-chromen-4-yl)-3H-benzo[f]chromen-3-one
CNP0232503.0	O=C(COc1cc(O)c2c(=O)c3ccccc3oc2c1)c1ccc(Br)cc1	3-[2-(4-bromophenyl)-2-oxoethoxy]-1-hydroxy-9H-xanthen-9-one
CNP0161305.0	O=c1cc(-c2cc3c(ccc4ccccc43)oc2=O)c2ccc(O)cc2o1	2-(7-hydroxy-2-oxochromen-4-yl)benzo[f]chromen-3-one
CNP0215076.0	CC(C)=CCc1cc2c(=O)cc(-c3ccc4c(c3)OCO4)oc2cc1O	3',4'-Methylenedioxy-7-hydroxy-6-isopentenyl flavone
CNP0158743.0	COc1ccc(-c2oc3c(ccc4ccccc43)c(=O)c2O)cc1OC	2-(3,4-dimethoxyphenyl)-3-hydroxybenzo[h]chromen-4-one
CNP0060882.0	COc1cccc2cc(-c3cc(=O)oc4cc(C)c(O)cc34)oc12	MLS001167538
CNP0307902.0	COc1cc2oc3c(OC)cccc3c(=O)c2c(O)c1CC=C(C)C	1-Hydroxy-3,5-dimethoxy-2-prenylxanthone
CNP0179234.0	O=C(COc1cc(O)c2c(=O)c3ccccc3oc2c1)c1ccccc1	1-hydroxy-3-(2-oxo-2-phenylethoxy)-9H-xanthen-9-one
CNP0257604.0	COc1ccc(-c2cc3cc(O)c(OC)cc3o2)c(OC)c1	Methylsainfuran
CNP0237607.0	COc1c(OCC=C(C)C)cc2c(c1O)c(=O)c1ccccc1n2C	Evoprenine
CNP0107936.0	COc1cccc2c1C(=O)c1c(cc3cc(C)cc(OC)c3c1O)C2=O	XR-651
CNP0290659.0	COc1ccc2c(c1)oc1c3ccc(O)cc3oc(=O)c21	9-O-Methylcoumestrol
CNP0295212.0	CC(=O)c1cc2c(cc(O)c3c(=O)c4ccccc4n(C)c32)o1	Hallacridone
CNP0164085.0	COc1c(O)c(OC)c2c(oc3ccccc32)c1OC	alpha-Pyrufuran
CNP0380553.0	COc1c(O)c(OC)c2oc3ccccc3c2c1OC	beta-Pyrufuran
CNP0258	COc1ccc(-c2cc3ccc(O)cc3oc2=O)cc1OC	3-(3,4-dimethoxyphenyl)-7-hydroxy-

108.0		2H-chromen-2-one
CNP0342494.0	COc1ccc(-c2cc3ccc(O)cc3oc2=O)c(OC)c1	3-(2,4-dimethoxyphenyl)-7-hydroxy-2H-chromen-2-one
CNP0120005.0	COc1ccc(OC)c(-c2cc3ccc(O)cc3oc2=O)c1	3-(2,5-dimethoxyphenyl)-7-hydroxy-2H-chromen-2-one
CNP0056776.0	COc1ccc2c(=O)c(O)c(-c3ccccc3OC)oc2c1	7,2'-dimethoxy-3-hydroxyflavone
CNP0114476.0	COc1cc(OC)c2c(-c3ccc(O)cc3)cc(=O)oc2c1	NSC634747
CNP0393445.0	COc1ccc(-c2cc(=O)c3cc(O)ccc3o2)cc1OC	2-(3,4-dimethoxyphenyl)-6-hydroxy-4H-chromen-4-one
CNP0226960.0	COc1cccc(-c2oc3ccccc3c(=O)c2O)c1OC	2',3'-Dimethoxy-3-hydroxyflavone
CNP0242739.0	COc1ccc(-c2cc(=O)c3ccc(O)cc3o2)cc1OC	2-(3,4-Dimethoxyphenyl)-7-hydroxy-4H-chromen-4-one
CNP0151289.0	COc1ccc(-c2coc3cc(O)ccc3c2=O)c(OC)c1	2'-METHOXYFORMONETIN
CNP0230428.0	COc1cc(OC)c2c(=O)c(-c3ccc(O)cc3)coc2c1	3-(4-hydroxyphenyl)-5,7-dimethoxy-4H-chromen-4-one
CNP0204308.0	COc1c(-c2ccccc2)oc2cc(O)cc(OC)c2c1=O	7-hydroxy-3,5-dimethoxyflavone
CNP0214392.0	COc1c(O)cc2oc(-c3ccccc3)cc(=O)c2c1OC	7-Hydroxy-5,6-Dimethoxyflavone
CNP0303046.0	COc1c(OC)c2c3c(nccc3c1O)C(=O)c1ccccc1-2	7H-Dibenzo(de,g)quinolin-7-one, 3-hydroxy-1,2-dimethoxy-
CNP0234343.0	COc1ccc(-c2ccc3c(OC)c(O)c(=O)cc-3o2)cc1	DTXSID901318675
CNP0396936.0	COc1ccc(-c2cc(=O)c3cc(N)ccc3o2)cc1OC	6-amino-2-(3,4-dimethoxyphenyl)chromen-4-one
CNP0363751.0	O=c1oc2cc(O)ccc2cc1-c1ccc2c(c1)OCO2	3-(benzo[d][1,3]dioxol-5-yl)-7-hydroxy-2H-chromen-2-one
CNP0403522.0	COc1cc2c(=O)c3ccccc3oc2c(OC)c1O	9H-Xanthen-9-one, 3-hydroxy-2,4-dimethoxy-
CNP0111095.0	COc1c(O)cc2c(=O)c3ccccc3oc2c1OC	2-hydroxy-3,4-dimethoxyxanthen-9-one
CNP0199031.0	COc1cc(O)c2c(=O)c3ccccc3n(C)c2c1OC	1-Hydroxy-3,4-dimethoxy-10-methylacridan-9-one
CNP0304781.0	O=c1c2ccccc2oc2cc(OCc3ccc(Br)cc3)cc(O)c12	3-[(4-bromobenzyl)oxy]-1-hydroxy-9H-xanthen-9-one
CNP0498187.0	O=c1c2ccccc2oc2cc(OCc3ccc(Cl)cc3)cc(O)c12	3-[(4-chlorobenzyl)oxy]-1-hydroxy-9H-xanthen-9-one
CNP0038310.0	CCOC(=O)c1c(-c2ccccc2)oc2cc(Br)c(O)cc12	ethyl 6-bromo-5-hydroxy-2-phenyl-1-benzofuran-3-carboxylate

CNP0386971.0	CCCc1cc(-c2oncc2-c2ccccc2)c(O)cc1OC	Oprea1_032730
CNP0387281.0	O=c1cc(-c2cc3cc(Br)ccc3o2)c2cc(O)ccc2o1	4-(5-bromo-1-benzofuran-2-yl)-6-hydroxy-2H-chromen-2-one
CNP0117000.0	O=c1cc(-c2cc3ccccc3o2)c2cc(O)ccc2o1	4-(1-benzofuran-2-yl)-6-hydroxychromen-2-one
CNP0171788.0	COc1ccc2cc(-c3ccc(O)cc3OC)oc2c1	Vignafuran
CNP0104473.1	Oc1ccc2c(c1)O[C@H]1c3cc4ccoc4cc3OC[C@@H]21	Neodunol
CNP0294749.0	COc1cc(OC)c2c(ccc3cc(O)c(OC)cc32)c1	Isobatatasin I
CNP0294742.0	COc1ccccc1C=CCc1ccc(O)c(OC)c1OC	DTXSID00702714
CNP0369477.0	COc1ccc(-c2cc(=O)oc3c(C)c(O)ccc23)cc1	7-hydroxy-4-(4-methoxyphenyl)-8-methyl-2H-chromen-2-one
CNP0398849.0	COc1ccccc1-c1c(C)oc2cc(O)ccc2c1=O	7-hydroxy-3-(2-methoxyphenyl)-2-methyl-4H-chromen-4-one
CNP0164438.0	COc1ccc(-c2oc3ccc(C)cc3c(=O)c2O)cc1	3-Hydroxy-2-(4-methoxyphenyl)-6-methyl-4H-chromen-4-one
CNP0465781.0	COc1ccc2c(O)c(C(=O)c3ccccc3)oc2c1	(2E)-2-[hydroxy(phenyl)methylidene]-6-methoxy-1-benzofuran-3-one
CNP0189405.0	COc1cc(O)cc(OC)c1C(=O)C=Cc1ccccc1	1-(4-hydroxy-2,6-dimethoxyphenyl)-3-phenylprop-2-en-1-one
CNP0378368.0	COc1ccc2oc(=O)c(-c3ccc(O)cc3)cc2c1	3-(4-hydroxyphenyl)-6-methoxy-2H-chromen-2-one
CNP0370932.0	COc1ccc2c(O)c(-c3ccccc3)c(=O)oc2c1	4-Hydroxy-7-methoxy-3-phenylcoumarin
CNP0307904.0	COc1ccc2oc(-c3ccc(O)cc3)cc(=O)c2c1	4'-HYDROXY-6-METHOXYFLAVONE
CNP0304478.0	COc1ccc2c(=O)c(O)c(-c3ccccc3)oc2c1	7-Methoxyflavonol
CNP0310594.0	COc1ccc(-c2cc(=O)c3cc(O)ccc3o2)cc1	6-Hydroxy-4'-methoxyflavone
CNP0024170.0	COc1ccccc1-c1cc(=O)c2cc(O)ccc2o1	6-Hydroxy-2'-methoxyflavone
CNP0046882.0	COc1ccccc1-c1cc(=O)c2ccc(O)cc2o1	7-HYDROXY-2'-METHOXYFLAVONE
CNP0334879.0	COc1ccccc1-c1coc2cc(O)ccc2c1=O	7-hydroxy-3-(2-methoxyphenyl)-4H-chromen-4-one
CNP0383696.0	COc1cccc(-c2cc(=O)c3c(O)cccc3o2)c1	5-HYDROXY-3'-METHOXYFLAVONE
CNP0142	COc1cc2c(=O)cc(-c3ccccc3)oc2cc1O	Trigraecum

696.0		
CNP0376297.0	COc1cc2c(=O)c(-c3ccccc3)coc2cc1O	7-hydroxy-6-methoxyisoflavone
CNP0314189.0	COc1ccccc1-c1cc(=O)c2c(O)cccc2o1	5-hydroxy-2'-methoxyflavone
CNP0068945.0	COc1cccc2oc(-c3ccc(O)cc3)cc(=O)c12	4'-hydroxy-5-methoxyflavone
CNP0321571.0	COc1cccc2oc(-c3ccccc3)c(O)c(=O)c12	3-HYDROXY-5-METHOXYFLAVONE
CNP0189987.0	CC(=O)c1cc2c(oc(=O)c3ccccc32)c(C)c1O	2-acetyl-3-hydroxy-4-methyl-6H-benzo[c]chromen-6-one
CNP0304550.0	Cn1c2ccccc2c(=O)c2c(O)cc3occc3c21	Furofoline I
CNP0048095.0	CC(=O)Oc1ccc2oc(-c3ccccc3)c(C(N)=O)c2c1	3-carbamoyl-2-phenyl-1-benzofuran-5-yl acetate
CNP0548172.0	COc1ccc(-c2cc(=O)c3cc(N)ccc3o2)cc1	6-amino-2-(4-methoxyphenyl)-4H-chromen-4-one
CNP0505568.0	COc1cccc(-c2cc(=O)c3ccccc3o2)c1N	167869-21-8
CNP0037689.0	O=Cc1c(O)ccc2c(=O)c(-c3ccccc3)coc12	7-hydroxy-4-oxo-3-phenyl-4H-chromene-8-carbaldehyde
CNP0370072.0	COc1ccc2c(c1)c(=O)oc1c(C)c(O)ccc12	3-hydroxy-8-methoxy-4-methyl-6H-benzo[c]chromen-6-one
CNP0264848.0	CC(C)(N)c1cc2cc3ccc(=O)oc3cc2o1	Prangosine
CNP0190090.0	COc1cccc2c(=O)c3cc(O)ccc3oc12	9H-Xanthen-9-one, 2-hydroxy-5-methoxy-
CNP0283178.0	COc1cc2c(=O)c3ccccc3oc2cc1O	3-hydroxy-2-methoxyxanthen-9-one
CNP0346206.0	COc1cccc2c(=O)c3ccc(O)cc3oc12	3-hydroxy-5-methoxyxanthen-9-one
CNP0358567.0	COc1ccc2c(=O)c3c(O)cccc3oc2c1	1-hydroxy-6-methoxyxanthen-9-one
CNP0348188.0	COc1c(O)ccc2c(=O)c3ccccc3oc12	3-Hydroxy-4-methoxyxanthone
CNP0105548.0	COC(=O)c1nccc2c1[nH]c1cc(OC)ccc12	Harmic Acid Methyl Ester
CNP0301984.0	COc1cccc2c1C(=O)c1ccc(C)c(O)c1C2=O	1-Hydroxy-5-methoxy-2-methylanthracene-9,10-dione
CNP0264940.0	COc1c(C)ccc2c1C(=O)c1ccc(O)cc1C2=O	6-Hydroxy-1-methoxy-2-methylanthracene-9,10-dione
CNP0140866.0	COc1cc(C)cc2c1C(=O)c1c(O)cccc1C2=O	8-Hydroxy-1-methoxy-3-methylanthraquinone

CNP0142337.0	COC(=O)c1cc2c([nH]c3ccccc32)c(C=O)n1	methyl 1-formyl-9H-pyrido[3,4-b]indole-3-carboxylate
CNP0586405.0	COc1cccc2c1C(=O)c1cccc(O)c1C2=O	1-Hydroxy-5-methoxyanthraquinone
CNP0337562.0	COc1cc(O)c2c(c1)C(=O)c1ccccc1C2=O	1-Hydroxy-3-methoxyanthracene-9,10-dione
CNP0277104.0	COc1cc(O)cc2c1C(=O)c1ccccc1C2=O	1-Methoxy-3-hydroxy-9,10-anthracenedione
CNP0121690.0	CNC1=C(Cl)C(=O)c2ccncc2C1=O	Caulibugulone C
CNP0355590.0	O=C1C(=Cc2ccc(-c3ccccc3)cc2)Oc2cc(O)ccc21	2-([1,1'-biphenyl]-4-ylmethylene)-6-hydroxybenzofuran-3(2h)-one
CNP0107149.0	O=c1cc(-c2ccc(O)cc2)oc2c1ccc1ccccc12	4H-Naphtho[1,2-b]pyran-4-one, 2-(4-hydroxyphenyl)-
CNP0051328.0	Cc1c(-c2ccc(O)cc2)c(=O)oc2ccc(Br)cc12	6-bromo-3-(4-hydroxyphenyl)-4-methylchromen-2-one
CNP0256539.0	Cc1c(-c2ccc(Br)cc2)c(=O)oc2cc(O)ccc12	3-(4-bromophenyl)-7-hydroxy-4-methyl-2H-chromen-2-one
CNP0353284.0	CC(C)c1ccc(-c2cc(=O)c3cc(N)ccc3o2)cc1	6-amino-2-[4-(propan-2-yl)phenyl]-4H-chromen-4-one
CNP0238414.0	CC(=O)c1cc2c(cc1O)oc1c(C)ccc(C)c12	Ruscodibenzofuran
CNP0354783.0	O=c1oc2cc(O)ccc2cc1-c1cccc(Br)c1	3-(3-bromophenyl)-7-hydroxychromen-2-one
CNP0253638.0	COc1cc2c(c3[nH]c4ccccc4c(=O)c13)C=CC(C)(C)O2	6-methoxy-3,3-dimethyl-12H-pyrano[2,3-c]acridin-7-one
CNP0128432.0	O=c1oc2cc(O)ccc2cc1-c1ccc(Cl)cc1	3-(4-chlorophenyl)-7-hydroxy-2H-chromen-2-one
CNP0489442.0	O=c1cc(-c2ccccc2)c2cc(Cl)c(O)cc2o1	6-chloro-7-hydroxy-4-phenyl-2H-chromen-2-one
CNP0369987.0	O=c1c(-c2ccccc2Cl)coc2cc(O)ccc12	3-(2-chlorophenyl)-7-hydroxy-4H-chromen-4-one
CNP0272462.0	COc1ccc2oc(-c3ccccc3)c(C(=O)O)c2c1	5-methoxy-2-phenyl-1-benzofuran-3-carboxylic acid
CNP0569182.0	CC(=O)c1c(C)oc2c1cc(O)c1ccccc12	1-(5-hydroxy-2-methylnaphtho[1,2-b]furan-3-yl)ethanone
CNP0513017.0	CCc1cc2c(-c3ccccc3)cc(=O)oc2cc1O	6-ethyl-7-hydroxy-4-phenyl-2H-chromen-2-one
CNP0227343.0	COc1cc(OC)c2c(ccc3ccc(O)cc32)c1	5,7-Dimethoxy-3-hydroxyphenanthrene
CNP0168021.0	COc1cc(OC)c2c(ccc3cc(O)ccc32)c1	5,7-DIMETHOXYPHENANTHREN-2-OL
CNP0145848.0	COc1ccc2c(ccc3cc(O)c(OC)cc32)c1	3,7-DIMETHOXYPHENANTHREN-2-OL

CNP0327553.0	COc1c(O)ccc(CC=Cc2ccccc2)c1OC	4-cinnamyl-2,3-dimethoxyphenol
CNP0243868.0	C=CC#Cc1ccc(-c2ccc(CO)s2)s1	(5'-(but-3-en-1-yn-1-yl)-[2,2'-bithiophen]-5-yl)methanol
CNP0366709.0	Cc1c(-c2ccccc2)c(=O)oc2ccc(O)cc12	6-hydroxy-4-methyl-3-phenyl-2H-chromen-2-one
CNP0313165.0	Cc1c(O)ccc2c(-c3ccccc3)cc(=O)oc12	7-hydroxy-8-methyl-4-phenyl-2H-chromen-2-one
CNP0343832.0	Cc1ccc2oc(-c3cccc(O)c3)cc(=O)c2c1	3'-HYDROXY-6-METHYLFLAVONE
CNP0251749.0	Cc1ccc2oc(-c3ccc(O)cc3)cc(=O)c2c1	4'-Hydroxy-6-methylflavone
CNP0237313.0	Cc1cc(O)c2c(-c3ccccc3)cc(=O)oc2c1	5-hydroxy-7-methyl-4-phenyl-2H-chromen-2-one
CNP0036846.0	Cc1c(-c2ccccc2)oc2cc(O)ccc2c1=O	7-Hydroxy-3-methylflavone
CNP0602040.0	O=C1c2ccccc2C(=O)c2c1ccc1c(=O)c3ccccc3[nH]c21	Naphth[2,3-c]acridine-5,8,14(13H)-trione
CNP0212301.0	Cc1ccccc1-c1cc(=O)c2cc(N)ccc2o1	6-amino-2-(2-methylphenyl)-4H-chromen-4-one
CNP0033405.0	O=C1c2ccccc2C(=O)c2c1cc1ccccc1c2O	6-hydroxytetracene-5,12-dione
CNP0065874.0	COc1cccc(C=CC(=O)c2ccccc2)c1O	2-HYDROXY-3-METHOXYCHALCONE
CNP0598187.0	COc1cc(OC)c2c(=O)c(-c3ccccc3)c[nH]c2c1	5,7-Dimethoxy-3-phenylquinolin-4(1H)-one
CNP0394042.0	O=c1oc2cc(O)ccc2cc1-c1ccccc1	7-hydroxy-3-phenyl-2H-chromen-2-one
CNP0313286.0	COc1ccc2c(c1)[nH]c1cc(OC)c(C=O)cc12	9H-Carbazole-3-carboxaldehyde, 2,7-dimethoxy-
CNP0165864.0	COc1ccc2[nH]c3c(OC)cc(C=O)cc3c2c1	1,6-Dimethoxy-9H-carbazole-3-carboxaldehyde
CNP0178326.0	COc1cc2c3c(c1)OCc1cc(O)cc(c1-3)CC2	Isoflavidinin
CNP0341626.0	Nc1c(-c2ccccc2)oc2ccccc2c1=O	3-Amino-2-phenyl-4H-chromen-4-one
CNP0379162.0	O=C1C(=Cc2ccccc2)Oc2cc(O)ccc21	2-benzylidene-6-hydroxy-1-benzofuran-3-one
CNP0398255.0	Cc1cc(O)c2c(=O)c3ccccc3oc2c1	1-hydroxy-3-methylxanthen-9-one
CNP0171398.0	COc1ccc2c(c1)[nH]c1c(C(C)=O)nccc12	arenarine c
CNP0173329.0	Cc1cc2c(c(O)c1C)C(=O)c1ccccc1C2=O	1-hydroxy-2,3-dimethylanthracene-9,10-dione

CNP0282947.0	Cc1ccc2c(c1)C(=O)c1ccc(O)cc1C2=O	2-Hydroxy-6-methylanthracene-9,10-dione
CNP0148625.0	Cc1ccc2c(c1)C(=O)c1cccc(O)c1C2=O	1-hydroxy-6-methylanthracene-9,10-dione
CNP0499120.0	COC(=O)c1ccc(Br)cc1-[n+]1ccc2c(c1)[nH]c1cc(Br)ccc12	methyl 4-bromo-2-(7-bromo-9H-pyrido[3,4-b]indol-2-ium-2-yl)benzoate
CNP0050328.0	Oc1ccc2c(c1)oc1c(Cl)c(Cl)ccc12	6,7-Dichloro-3-dibenzofuranol
CNP0337989.0	O=C1c2ccccc2C(=Cc2ccc(O)cc2)c2ccccc21	10-(4-HYDROXYBENZYLIDENE)ANTHRACEN-9(10H)-ONE
CNP0277697.0	COc1cc2ccc3c(C)c(C)ccc3c2cc1O	2-Methoxy-7,8-dimethylphenanthren-3-ol
CNP0082191.0	Oc1ccc2oc3cc(Cl)ccc3c2c1	7-Chloro-2-dibenzofuranol
CNP0041429.0	COc1ccc2c(c1)CCC(c1ccc(O)cc1)=C2	p-(3,4-Dihydro-6-methoxy-2-naphthyl)phenol
CNP0490987.0	O=C1c2ccccc2-c2ccc(O)c3cccc1c23	3-Hydroxybenz(de)anthracen-7-one
CNP0353633.0	COc1cc2[nH]c3c(OC)cccc3c2cc1C	1,7-DIMETHOXY-6-METHYL-9H-CARBAZOLE
CNP0274647.0	COc1ccc2c(c1)[nH]c1cc(OC)ccc12	2,7-dimethoxy-9h-carbazole
CNP0436922.0	O=C1C(c2ccccc2)=C(O)c2ccccc21	3-hydroxy-2-phenyl-1H-inden-1-one
CNP0167781.0	COc1ccc2[nH]c3ccc(C=O)cc3c2c1	6-methoxy-9H-carbazole-3-carbaldehyde
CNP0129523.0	COc1cnc(C)c2[nH]c3ccccc3c12	4-Methoxy-1-methyl-9h-pyrido[3,4-b]indole
CNP0249827.0	NC(=CC(=O)c1ccccc1)c1ccccc1	3-amino-1,3-diphenylprop-2-en-1-one
CNP0172388.0	O=Cc1nccc2c1[nH]c1ccccc12	9H-pyrido[3,4-b]indole-1-carbaldehyde
CNP0321303.0	Oc1cccc2c1ccc1cc3ccccc3cc12	Benz(a)anthracen-4-ol
CNP0377340.0	CC(C)=Cc1nccc2c1[nH]c1ccccc12	1-(2-methyl-1-propen-1-yl)-9h-pyrido[3,4-b]indole
CNP0524542.0	O=C1c2ccc(Br)cc2-[n+]2ccc3c([nH]c4ccccc43)c21	16-bromo-3-aza-13-azoniapentacyclo[11.7.0.02,10.04,9.014,19]icosa-1(13),2(10),4,6,8,11,14(19),15,17-nonaen-20-one
CNP0294	CC=C(c1ccccc1)c1ccccc1N	2-(1-Phenyl-1-propenyl)aniline

065.0		
CNP0210 210.0	Oc1cc2ccccc2c2ccccc12	Phenanthren-9-ol
CNP0194 305.0	CC(=O)c1cccc2c1[nH]c1ccccc12	1-(9H-carbazol-1-yl)ethanone
CNP0526 486.0	COc1ccc2[nH]c3ccccc3c2c1	3-Methoxy-9H-carbazole
CNP0244 552.0	O=C1CCCCc2c1[nH]c1ccccc21	7,8,9,10-tetrahydrocyclohepta[b]indol-6(5H)-one
CNP0273 090.0	CC(C)=CC=C1C(=O)Nc2ccccc21	3-(3-methylbut-2-enylidene)-1H-indol-2-one
CNP0115 557.0	CC=C1C(=O)Nc2ccccc21	3-ethylidene-1H-indol-2-one
CNP0284 140.0	COC(=O)C(=Cc1ccc2c(c1)OCO2)C(=Cc1ccc2c(c1)OCO2)C(=O)OC	DIMETHYL (2Z,3Z)-2,3-BIS(BENZO[1,3]DIOXOL-5-YLMETHYLIDENE)BUTANEDIOATE
CNP0411 665.0	COc1cc(-c2cc(=O)c3ccc(OC)c(OC)c3o2)cc(OC)c1OC	7,8,3',4',5'-pentamethoxyflavone
CNP0294 570.0	COc1cc(OC)c2c(=O)cc(-c3ccc(OC)c(OC)c3OC)oc2c1	2',3',4',5,7-Pentamethoxyflavone
CNP0244 190.0	COc1cc2occ(-c3ccc4c(c3)OCO4)c(=O)c2c(OC)c1OC	5,6,7-Trimethoxy-3',4'-methylenedioxyisoflavone
CNP0276 279.0	COc1cc(OC)c2c(=O)cc(-c3cc(OC)c4c(c3)OCO4)oc2c1	5,7,3'-Trimethoxy-4',5'-methylenedioxyflavone
CNP0168 871.0	COc1ccc2oc3c(OC)c(OC)c(OC)c(OC)c3c(=O)c2c1	1,2,3,4,7-PENTAMETHOXY-9H-XANTHEN-9-ONE
CNP0381 357.0	CC(=O)Oc1ccc2c(-c3cc4c(ccc5ccccc54)oc3=O)cc(=O)oc2c1C	8-methyl-2-oxo-4-(3-oxo-3H-benzo[f]chromen-2-yl)-2H-chromen-7-yl acetate
CNP0380 708.0	CC(=O)Oc1ccc2c(-c3cc4c(ccc5ccccc54)oc3=O)cc(=O)oc2c1	2-oxo-4-(3-oxo-3H-benzo[f]chromen-2-yl)-2H-chromen-7-yl acetate
CNP0106 626.0	COc1cccc2cc(-c3cc(=O)oc4ccc(OC(C)=O)cc34)oc12	4-(7-methoxy-1-benzofuran-2-yl)-2-oxo-2H-chromen-6-yl acetate
CNP0316 309.0	COc1cc2occc2c2occ(-c3ccc4c(c3)OCO4)c(=O)c12	Garhwalin
CNP0247 123.0	C=CC(C)(C)Oc1c(OC)c(=O)oc2cc3occc3c(OC)c12	Halfordinin
CNP0192 225.0	COc1ccc(-c2coc3c(OC)c(OC)ccc3c2=O)c(OC)c1	7,8,2',4'-Tetramethoxy-isoflavone
CNP0055 996.0	COc1cc(OC)c2c(=O)c(-c3ccc(OC)c(OC)c3)coc2c1	5,7,3',4'-Tetramethoxyisoflavone
CNP0184	COc1cc(OC)cc(-c2cc(=O)c3c(OC)c(OC)ccc3o2)c1	Cerrosillin

672.0		
CNP0311 309.0	COc1cc(OC)c2c(=O)c(OC)c(-c3ccccc3OC)oc2c1	Flavone, 2',3,5,7-tetramethoxy-
CNP0185 907.0	COc1ccc(-c2coc3cc(OC)c(OC)c(OC)c3c2=O)cc1	5,6,7,4'-Tetramethoxyisoflavone
CNP0198 406.0	COc1ccc(-c2coc3cc4c(cc3c2=O)OCO4)cc1OC	3',4'-Dimethoxy-6,7-methylenedioxyisoflavone
CNP0127 198.0	COc1ccc2c(=O)c(-c3cc4c(cc3OC)OCO4)coc2c1	Cuneatin methyl ether
CNP0266 791.0	COc1cc(OC)c2c(OC)c3ccoc3nc2c1OC	Acronycidine
CNP0372 257.0	COc1cc(OC)c2c(=O)c3cc(OC)c(OC)cc3oc2c1	1,3,6,7-Tetramethoxyxanthen-9-one
CNP0111 544.0	COc1cc2oc3c(OC)cccc3c(=O)c2c(OC)c1OC	1,2,3,5-tetramethoxyxanthen-9-one
CNP0285 040.0	COc1ccc2oc3cc4c(c(OC)c3c(=O)c2c1)OCO4	1,7-Dimethoxy-2,3-methylenedioxyxanthone
CNP0190 550.0	COc1cc(=O)oc(C=Cc2ccc3c(c2)OCO3)c1OC	AGN-PC-0JU150
CNP0250 284.0	O=c1oc2cc3occ(-c4ccc5c(c4)OCO5)c3cc2c2c1CCCC2	10-(1,3-benzodioxol-5-yl)-1,2,3,4-tetrahydro-5H-benzo[c]furo[3,2-g]chromen-5-one
CNP0047 198.0	COc1cc(-c2cc(=O)c3cc(Cl)cc(Cl)c3o2)cc(OC)c1OC	6,8-dichloro-2-(3,4,5-trimethoxyphenyl)-4H-chromen-4-one
CNP0132 814.0	COc1ccc(-c2coc3cc4c(c(OC)c3c2=O)C=CC(C)(C)O4)cc1	Alpinumisoflavone dimethyl ether
CNP0212 172.0	Cc1ccc2oc(-c3cc(=O)oc4cc5c(cc34)OCO5)c(C)c2c1	8-(3,5-dimethyl-1-benzofuran-2-yl)-6H-[1,3]dioxolo[4,5-g]chromen-6-one
CNP0326 071.0	COc1ccc(-c2cc3cc4c(C)coc4cc3oc2=O)cc1OC	6-(3,4-dimethoxyphenyl)-3-methyl-7H-furo[3,2-g]chromen-7-one
CNP0159 772.0	CC(=O)Oc1cc2oc3cc(=O)c4ccccc4c-3nc2c2ccccc12	5-oxo-5H-dibenzo[a,j]phenoxazin-9-yl acetate
CNP0365 984.0	O=c1oc2cc3occ(-c4ccc5c(c4)OCO5)c3cc2c2c1CCC2	9-(1,3-benzodioxol-5-yl)-2,3-dihydrocyclopenta[c]furo[3,2-g]chromen-4(1H)-one
CNP0346 522.0	COc1cc2cnc3c4cc5c(cc4ccc3c2cc1OC)OCO5	NSC166719
CNP0268 326.0	C=CC(C)(C)c1cc2c(OC)c3ccoc3c(OC)c2oc1=O	5,8-Dimethoxychalepensin
CNP0384 118.0	O=c1cc(-c2cc3ccccc3o2)c2cc3c(cc2o1)OCO3	8-(benzofuran-2-yl)-6H-[1,3]dioxolo[4,5-g]chromen-6-one
CNP0210	c1cc2cc3c(cc2o1)OC[C@@H]1c2cc4c(cc2O[C@H]31)OCO	NSC356827

809.1	4	
CNP0169<br>493.0	COc1cc(C=O)cc2cc(-c3ccc4c(c3)OCO4)oc12	DB-133062
CNP0185<br>989.1	COc1c2c(nc3occc13)[C@@](CC=C(C)C)(OC)C(=O)C=C2	(R)-Perfamine
CNP0268<br>298.0	COc1ccc2cc(-c3ccc(OC)c(OC)c3)c(=O)oc2c1	3-(3,4-Dimethoxyphenyl)-7-methoxy-2H-chromen-2-one
CNP0108<br>983.0	COc1ccc(-c2cc3ccc(OC)cc3oc2=O)c(OC)c1	3-(2,4-dimethoxyphenyl)-7-methoxy-2H-chromen-2-one
CNP0369<br>436.0	COc1ccc2oc(-c3ccc(OC)c(OC)c3)cc(=O)c2c1	2-(3,4-dimethoxyphenyl)-6-methoxy-4H-chromen-4-one
CNP0371<br>741.0	COc1ccc(-c2cc(=O)oc3c(OC)c(OC)ccc23)cc1	7,8-dimethoxy-4-(4-methoxyphenyl)-2H-chromen-2-one
CNP0252<br>225.0	COc1ccc(-c2cc(=O)oc3cc(OC)cc(OC)c23)cc1	5,7,4'-Trimethoxy-4-phenylcoumarin
CNP0063<br>760.0	COc1ccc2c(=O)cc(-c3cccc(OC)c3OC)oc2c1	7,2',3'-Trimethoxyflavone
CNP0184<br>519.0	COc1cc(OC)c2c(=O)cc(-c3ccccc3OC)oc2c1	5,7,2'-Trimethoxyflavone
CNP0268<br>633.0	COc1ccccc1-c1cc(=O)c2c(OC)c(OC)ccc2o1	5,6,2'-Trimethoxyflavone
CNP0402<br>662.0	COc1cc(OC)c2c(=O)c(-c3ccccc3OC)coc2c1	5,7-dimethoxy-3-(2-methoxyphenyl)-4H-chromen-4-one
CNP0287<br>519.0	COc1cc2occ(-c3ccccc3)c(=O)c2c(OC)c1OC	DTXSID001244825
CNP0176<br>370.0	COc1cc(OC)c2c(=O)cc(-c3ccccc3)oc2c1OC	Norwogonin 5,7,8-trimethyl ether
CNP0513<br>552.0	COc1ccccc1-c1coc2cc(OC(C)=O)ccc2c1=O	3-(2-methoxyphenyl)-4-oxo-4h-chromen-7-yl acetate
CNP0389<br>167.0	COc1cc2oc3ccccc3c(=O)c2c(OC)c1OC	1,2,3-trimethoxyxanthen-9-one
CNP0396<br>557.0	COc1c(OC)c2cc3ccoc3cc2oc1=O	Halkendin
CNP0242<br>850.0	COn1c(=O)cc2c3ccccc3n3c(=O)ccc1c23	3H-Indolo(3,2,1-de)(1,5)naphthyridine-2,6-dione, 3-methoxy-
CNP0576<br>483.0	O=C1c2ccccc2C(=O)c2c1oc(-c1ccccc1)c2C(=O)c1ccccc1	3-benzoyl-2-phenylnaphtho[2,3-b]furan-4,9-dione
CNP0407<br>785.0	COc1ccc(-c2cc(=O)oc3c(C)c4occ(C)c4cc23)cc1	5-(4-methoxyphenyl)-3,9-dimethyl-7H-furo[3,2-g]chromen-7-one
CNP0374<br>855.0	COc1cccc(-c2coc3c2c(C)cc2oc(=O)cc(C)c23)c1	3-(3-methoxyphenyl)-4,9-dimethyl-7H-furo[2,3-f]chromen-7-one
CNP0374<br>555.0	COc1ccc(-c2coc3c2c(C)cc2oc(=O)cc(C)c23)cc1	3-(4-methoxyphenyl)-4,9-dimethyl-7H-furo[2,3-f]chromen-7-one

CNP0151514.0	CC(=O)c1oc2ccc3c(C)cc(=O)oc3c2c1-c1ccccc1	8-acetyl-4-methyl-9-phenyl-2H-furo[2,3-h]chromen-2-one
CNP0363477.0	O=C(Oc1c(-c2ccccc2)oc2ccccc2c1=O)c1ccccc1	3-Benzoyloxy flavone
CNP0197687.0	COc1ccc(-c2coc3cc4oc(=O)cc(C)c4cc23)cc1	3-(4-methoxyphenyl)-5-methyl-7H-furo[3,2-g]chromen-7-one
CNP0393499.0	COc1ccc(-c2cc3cc4c(C)coc4cc3oc2=O)cc1	6-(4-methoxyphenyl)-3-methyl-7H-furo[3,2-g]chromen-7-one
CNP0127257.0	COc1cc2c(-c3cc4ccccc4o3)cc(=O)oc2cc1C	4-(1-benzofuran-2-yl)-6-methoxy-7-methyl-2H-chromen-2-one
CNP0367387.0	COc1ccc(-c2cc(=O)oc3cc4occ(C)c4cc23)cc1	5-(4-methoxyphenyl)-3-methyl-7H-furo[3,2-g]chromen-7-one
CNP0405773.0	COc1c2ccc(OCC=C(C)C)cc2nc2occc12	Furo(2,3-b)quinoline, 4-methoxy-7-((3-methyl-2-butenyl)oxy)-
CNP0048103.0	COc1ccc(-c2cc(=O)c3cc(Br)ccc3o2)cc1OC	6-bromo-2-(3,4-dimethoxyphenyl)-4H-chromen-4-one
CNP0330353.0	COc1ccc2oc(=O)cc(-c3cc4ccccc4o3)c2c1	4-(1-benzofuran-2-yl)-6-methoxy-2H-chromen-2-one
CNP0257212.0	COc1ccccc1-c1cc(=O)c2ccc3occc3c2o1	4H-Furo(2,3-h)-1-benzopyran-4-one, 2-(2-methoxyphenyl)-
CNP0028275.0	COc1ccc(-c2cc(=O)c3cc(Cl)ccc3o2)cc1OC	6-chloro-2-(3,4-dimethoxyphenyl)-4H-chromen-4-one
CNP0164706.0	C=CC(C)(C)c1c2ccoc2c(OC)c2oc(=O)ccc12	Benahorin
CNP0325873.0	C=CC(C)(C)c1c2occc2c(OC)c2ccc(=O)oc12	FUROPINNARIN
CNP0157494.0	COc1ccc2c(c1)OCc1c-2oc2cc(OC)ccc12	Dehydrovariabilin
CNP0342780.0	COc1ccc2c(c1)c(=O)oc1cc3occ(C)c3cc12	3-methoxy-10-methyl-5H-benzo[c]furo[3,2-g]chromen-5-one
CNP0130981.0	COc1ccc(C=Cc2cc(OC)c(OC)c(OC)c2)cc1	1,2,3-trimethoxy-5-[2-(4-methoxyphenyl)ethenyl]benzene
CNP0157521.0	CC(C)=CCOc1cc2occc2c2oc(=O)ccc12	LANATIN
CNP0254418.0	COc1cc2oc(-c3ccccc3)cc(=O)c2c(OC)c1C	Dimethylstrobochrysin
CNP0140232.0	COc1cc2ccc3cc(OC)cc4c(=O)oc(c1)c2c34	DTXSID701235192
CNP0371254.0	COc1ccc(-c2cc(=O)oc3cc(OC)ccc23)cc1	7-methoxy-4-(4-methoxyphenyl)-2H-chromen-2-one
CNP0135538.0	COc1ccc2oc(-c3ccccc3OC)cc(=O)c2c1	6,2'-dimethoxyflavone
CNP0256327.0	COc1ccc(OC)c(-c2cc(=O)c3ccccc3o2)c1	2',5'-DIMETHOXYFLAVONE

CNP0380472.0	COc1ccc2c(=O)c(-c3ccccc3OC)coc2c1	7-methoxy-3-(2-methoxyphenyl)-4H-chromen-4-one
CNP0365147.0	COc1ccc2c(-c3ccccc3)cc(=O)oc2c1OC	7,8-dimethoxy-4-phenyl-2H-chromen-2-one
CNP0044831.0	COc1ccc(-c2cc(=O)c3ccccc3o2)c(OC)c1	2',4'-dimethoxyflavone
CNP0264833.0	COc1ccc(-c2coc3cc(OC)ccc3c2=O)cc1	4',7-Dimethoxyisoflavone
CNP0241708.0	COc1cccc(-c2cc(=O)c3ccccc3o2)c1OC	2',3'-Dimethoxyflavone
CNP0125280.0	C=CC(C)(C)c1cc2ccc(OC)c(OC)c2oc1=O	3-(1,1-Dimethyl-2-propenyl)-7,8-dimethoxy-2H-1-benzopyran-2-one
CNP0372270.0	O=c1cc(-c2ccc3c(c2)OCO3)oc2ccccc12	2-(1,3-benzodioxol-5-yl)-4H-chromen-4-one
CNP0349838.0	COc1ccc2c(c1)oc(=O)c1cc(OC)ccc12	3,8-dimethoxybenzo[c]chromen-6-one
CNP0238573.0	COc1ccc(C=Cc2cc(OC)cc(=O)o2)cc1	Demethoxyyangonin;5,6-Dehydrokavain
CNP0257962.0	COc1cc(C)c(OC)c2c1C(=O)c1ccccc1C2=O	1,4-Dimethoxy-2-methylanthracene-9,10-dione
CNP0162582.0	CC(=O)Oc1cccc2nc3ccccc3nc12	phenazin-1-yl acetate
CNP0393628.0	COc1ccc2c(c1)C(=O)c1c-2ncc(OC)c1C	3,7-Dimethoxy-4-methyl-5H-indeno[1,2-b]pyridin-5-one
CNP0309184.0	COc1cccc2cc3c(c(OC)c12)C(=O)OC3	8,9-dimethoxynaphtho[2,3-c]furan-1(3H)-one
CNP0140258.0	COc1ccc2c(c1OC)C(=O)c1ccccc1C2=O	1,2-dimethoxyanthracene-9,10-dione
CNP0269607.0	CC=Cc1cc(OC)c2c(c1OC)OCO2	4,7-Dimethoxy-5-(prop-1-en-1-yl)-2H-1,3-benzodioxole
CNP0157821.0	COc1cccc2c(=O)c3ccoc3n(C)c12	8-Methoxy-9-methylfuro[2,3-b]quinolin-4(9H)-one
CNP0566278.0	O=C1c2ccccc2C(=O)c2c1ccc1c2OCCO1	1,2-ethylenedioxyanthraquinone
CNP0177321.0	O=c1cccc(-c2ccc3c(c2)OCO3)o1	Paracotoin
CNP0538528.0	O=c1ccc2cc3c(-c4ccc(Cl)cc4)coc3cc2o1	3-(4-chlorophenyl)-7H-furo[3,2-g]chromen-7-one
CNP0365745.0	Cc1coc2c(C)c3oc(=O)cc(-c4ccccc4)c3cc12	3,9-dimethyl-5-phenyl-7H-furo[3,2-g]chromen-7-one
CNP0136502.0	Cc1cc2oc(=O)cc(-c3cc4ccccc4o3)c2cc1C	4-(1-benzofuran-2-yl)-6,7-dimethyl-2H-chromen-2-one
CNP0351284.0	Cc1coc2c1c(C)cc1oc(=O)cc(-c3ccccc3)c12	3,4-dimethyl-9-phenyl-7H-furo[2,3-f]chromen-7-one

CNP0316707.0	COc1c(C(=O)C(C)=Cc2ccccc2)ccc2occc12	DTXSID401174771
CNP0045220.0	COc1ccc(-c2cc(=O)c3c(ccc4ccccc43)o2)cc1	4'-methoxy-5,6-benzoflavone
CNP0261258.0	COc1ccccc1-c1cc(=O)c2c(ccc3ccccc32)o1	3-(2-methoxyphenyl)benzo[f]chromen-1-one
CNP0251100.0	CC1(C)CCc2cc3c(-c4ccccc4)cc(=O)oc3cc2O1	8,8-Dimethyl-4-phenyl-7,8-dihydro-2H,6H-pyrano[3,2-g]chromen-2-one
CNP0191261.0	Cc1coc2cc3oc(=O)c(-c4ccccc4)cc3cc12	3-methyl-6-phenyl-7H-furo[3,2-g]chromen-7-one
CNP0184051.0	Cc1coc2cc3oc(=O)cc(-c4ccccc4)c3cc12	3-methyl-5-phenyl-7H-furo[3,2-g]chromen-7-one
CNP0257986.0	Cc1oc2cc3oc(=O)ccc3cc2c1-c1ccccc1	2-methyl-3-phenyl-7H-furo[3,2-g]chromen-7-one
CNP0177693.0	Cc1ccc(-c2coc3cc4oc(=O)ccc4cc23)cc1	3-(4-methylphenyl)-7H-furo[3,2-g]chromen-7-one
CNP0167313.0	COc1c(C(=O)C=Cc2ccccc2)ccc2occc12	DB-290762
CNP0365596.0	Cc1coc2c(C)c3oc(=O)c4ccccc4c3cc12	7,10-dimethyl-5H-benzo[c]furo[3,2-g]chromen-5-one
CNP0184828.0	O=C1OC(=O)c2c(-c3ccccc3)ccc3cccc1c23	4-Phenyl-1H,3H-naphtho[1,8-cd]pyran-1,3-dione
CNP0219495.0	COc1ccc(-c2coc3cc(C)ccc3c2=O)cc1	7-O-methylformononetin
CNP0231831.0	COc1cc(C)cc2oc(=O)cc(-c3ccccc3)c12	5-methoxy-7-methyl-4-phenyl-2H-chromen-2-one
CNP0261778.0	CC(C)c1cc2cc3ccc(=O)oc3cc2o1	2-isopropyl-7H-furo[3,2-g]chromen-7-one
CNP0267123.0	COc1ccc(-c2cc3ccccc3c(=O)o2)cc1	3-(4-methoxyphenyl)-1h-isochromen-1-one
CNP0316618.0	COc1ccc(-c2coc3ccccc3c2=O)cc1	4'-O-Methylisoflavone
CNP0114627.0	COc1c2c(nc3ccccc13)OC(C)(C)C=C2	5-Methoxy-2,2-dimethyl-2H-pyrano[2,3-b]quinoline
CNP0312596.0	COc1cc(=O)cc2oc(-c3ccccc3)ccc1-2	5-Methoxy-2-phenyl-7H-1-benzopyran-7-one
CNP0482029.0	COc1cc2ccccc2c2c(=O)cc(C)oc12	5-Methoxy-3-methyl-1H-naphtho[2,1-b]pyran-1-one
CNP0224693.0	Cc1oc2cc3oc(=O)ccc3cc2c1C	2,3-dimethylfuro[3,2-g]chromen-7-one
CNP0361061.0	O=C1c2ccccc2-c2oc(=O)c3ccccc3c21	Indeno[1,2-c]isochromene-5,11-dione
CNP0217	COc1ccc2oc3ccccc3c(=O)c2c1	2-Methoxyxanthen-9-one

069.0		
CNP0202868.0	COc1cccc2c(=O)c3ccccc3oc12	4-Methoxy-9H-xanthen-9-one
CNP0170864.0	C=Cc1nccc2c3ccccc3n(OC)c12	N-Methoxy-1-vinyl-beta-carboline
CNP0208229.0	Cc1cccc2oc3occc3c(=O)c12	5-Methyl-4H-furo[2,3-b][1]benzopyran-4-one
CNP0086265.0	O=C1OC(=O)C(c2ccccc2)=C1c1ccccc1	2,3-Diphenylmaleic anhydride
CNP0271775.0	COc1cccc2c(=O)c3c(oc12)CCCC3	MLS002920291
CNP0369028.0	COn1c2ccccc2c2cc(C=O)ccc21	N-methoxy-3-formylcarbazole
CNP0138868.0	CC(=O)C=CC=Cc1ccc2c(c1)OCO2	3.4-Methylendioxycinnamalaceton
CNP0095612.0	O=c1cc(-c2ccc(Br)cc2)oc2ccc(Br)cc12	6,4'-DIBROMOFLAVONE
CNP0324157.0	O=c1cc(-c2ccccc2)oc2c(Br)cc(Br)cc12	6,8-dibromoflavone
CNP0309065.0	COC1=Cc2cccc3ccc(-c4ccccc4)c(c23)C1=O	2-O-Methylanigorufone
CNP0108478.0	COc1c2cccc(C)c2c(C)c2c(C)coc12	O-Methylcacalodienol
CNP0221043.0	C=Cc1ccc(-c2ccc(C#CC)s2)s1	5-(1-Propynyl)-5'-vinyl-2,2'-bithiophene
CNP0412486.0	CC(C)=CC=Cc1ccc2c(c1)C(=O)c1ccccc1C2=O	DTXSID101216564
CNP0215121.0	C=CC#Cc1ccc(-c2ccc(C)s2)s1	2,2'-Bithiophene, 5-(3-buten-1-ynyl)-5'-methyl-
CNP0481548.0	O=c1cc2cccc3c4cccc5cccc(c(c1=O)c23)c54	Perylene-1,2-dione
CNP0146686.0	CC(C)=CCC1=C(C)C(=O)c2ccccc2C1=O	Lepachol acetate
CNP0598859.0	O=C1C=CC(=O)C(c2ccc(-c3ccccc3)cc2)=C1	2-(4-phenylphenyl)cyclohexa-2,5-diene-1,4-dione
CNP0400525.0	Cc1ccc2c(c1)C(=O)c1ccc(C)cc1C2=O	2,6-Dimethylanthraquinone
CNP0489833.0	Cc1ccc2c(c1C)C(=O)c1ccccc1C2=O	1,2-Dimethylanthraquinone
CNP0251681.0	COC1=Cc2cccc3cccc(c23)C1=O	2-methoxyphenalen-1-one
CNP0384074.0	CC1=Cc2cc3ccccc3cc2C(=O)C1=O	3-Methylanthracene-1,2-dione

CNP0204442.0	CC1=Cc2cccc(C)c2C(=O)C1=O	3,8-Dimethyl-1,2-naphthoquinone
CNP0542249.0	Cc1ccc(C2=CC(=O)C=CC2=O)cc1	2-(p-Tolyl)-p-benzoquinone
CNP0182457.0	O=C1C(c2ccccc2)=C(c2ccccc2)c2ccccc21	2,3-Diphenyl-1H-inden-1-one
CNP0367302.0	CC=C(C)n1c2ccccc2c2ccccc21	9H-Carbazole, 9-(methyl-1-propenyl)-
CNP0305170.0	Cc1ccc2c(C)cc3occ(C)c3c2c1	Dehydrochromolaenin
CNP0168308.0	O=C1CCCC(c2ccccc2)=C1c1ccccc1	2,3-diphenylcyclohex-2-en-1-one
CNP0318117.0	CC=Cn1c2ccccc2c2ccccc21	9-(1-Propenyl)carbazole
CNP0386340.0	Cc1ccc2c(c1)Oc1cc(C)ccc1C2	3,6-Dimethyl-9H-xanthene
CNP0401439.0	Cc1cc2cc3ccccc3cc2cc(C)c1=O	8H-Cyclohepta(b)naphthalen-8-one, 7,9-dimethyl-
CNP0066879.0	CC1=C(c2ccccc2)c2ccccc2C1=O	2-methyl-3-phenyl-1H-inden-1-one
CNP0182468.0	C1=Cc2ccccc2C1=C1C=Cc2ccccc21	3,3'-biindenyl
CNP0464916.0	C1=CC=CC=CC=CC=CC=CC=CC=C1	cyclohexadecaoctaene
CNP0300454.0	C=C(C)c1ccc(C)c2ccc(C)c-2c1	Lactarazulene
CNP0390064.0	C(C=Cc1ccccc1)=Cc1ccccc1	Benzene, 1,1'-(1,3-butadiene-1,4-diyl)bis-
CNP0314756.0	C=C(C)C1=Cc2c(C)cccc2CC1	ID4EE9VDS0
CNP0129214.0	Cc1cccc2c1Cc1c(C)cccc1-2	1,8-dimethyl-9H-fluorene
CNP0078609.0	C1=C(c2ccccc2)Cc2ccccc21	2-Phenyl-1H-indene
CNP0568511.0	C=C1c2ccccc2-c2ccccc21	9-methylidenefluorene
CNP0185507.0	Brc1ccc2c(c1)C=CC2	5-bromo-1H-indene
CNP0033512.0	O=C1C=Cc2ccccc2C1=O	1,2-NAPHTHOQUINONE
CNP0388462.0	O=C1C=CC(=O)c2ccccc21	1,4-NAPHTHOQUINONE
CNP0338363.0	COc1cccc2oc(-c3ccccc3)cc(=O)c12	5-Methoxyflavone

CNP0248340.0	COc1ccc(-c2cc(=O)c3c(O)cc(O)cc3o2)cc1	acacetin
CNP0192500.0	O=c1ccc2ccc3occc3c2o1	Angelicin
CNP0287592.0	O=C1c2ccccc2C(=O)c2ccccc21	ANTHRAQUINONE
CNP0251015.0	O=c1cc(-c2ccc(O)cc2)oc2cc(O)cc(O)c12	apigenin
CNP0329318.0	O=c1cc(-c2ccccc2)oc2cc(O)c(O)c(O)c12	baicalein
CNP0105179.0	O=Cc1ccccc1	benzaldehyde
CNP0190749.0	O=c1cc(-c2ccccc2)oc2ccc3ccccc3c12	beta-naphthoflavone
CNP0314566.0	COc1ccc(-c2coc3cc(O)cc(O)c3c2=O)cc1	biochanin A
CNP0210919.0	Cc1ccc(C(C)C)cc1O	CARVACROL
CNP0280624.0	O=c1cc(-c2ccccc2)oc2cc(O)cc(O)c12	chrysin
CNP0129787.0	COc1cc(-c2cc(=O)c3c(O)c(OC)c(OC)cc3o2)ccc1O	Cirsilineol
CNP0492502.0	Oc1cc(O)c2cc(O)c(-c3ccc(O)c(O)c3)[o+]c2c1	Cyanidin
CNP0246782.0	COc1ccc(-c2cc(=O)c3c(O)cc(O)cc3o2)cc1O	Diosmetin
CNP0156199.0	COc1ccc(-c2cc(=O)c3c(O)c(OC)c(O)cc3o2)cc1OC	Eupatilin
CNP0174866.0	O=c1c(-c2ccc(O)cc2)coc2cc(O)cc(O)c12	genistein
CNP0156838.0	COc1cc2c(=O)c(-c3ccc(O)cc3)coc2cc1O	Glycitein
CNP0286996.0	Cc1nccc2c1[nH]c1ccccc12	Harman
CNP0246516.0	Oc1ccc(O)cc1	hydroquinone
CNP0071161.0	O=C1C(c2[nH]c3ccccc3c2O)=Nc2ccccc21	INDIGO
CNP0106378.0	OCc1c[nH]c2ccccc12	Indole-3-carbinol
CNP0304008.0	COc1cc(-c2oc3cc(O)cc(O)c3c(=O)c2O)ccc1O	Isorhamnetin
CNP0292269.0	O=c1c(O)c(-c2ccc(O)cc2)oc2cc(O)cc(O)c12	kaempferol

CNP0333612.0	O=C(O)c1cc(=O)c2ccccc2[nH]1	kynurenic acid
CNP0279503.0	O=c1cc(-c2ccc(O)c(O)c2)oc2cc(O)cc(O)c12	luteolin
CNP0287207.0	Cc1occc(=O)c1O	MALTOL
CNP0192878.0	COc1c(O)cc2oc(-c3ccccc3)cc(=O)c2c1O	Oroxylin A
CNP0525213.0	Oc1ccc(-c2[o+]c3cc(O)cc(O)c3cc2O)cc1	Pelargonidin
CNP0299558.0	Oc1cc(O)cc(O)c1	phloroglucinol
CNP0293402.0	CC1=CC(=O)c2c(O)cccc2C1=O	Plumbagin
CNP0328096.0	O=c1ccc2cc3ccoc3cc2o1	Psoralen
CNP0103504.0	O=c1c(O)c(-c2ccc(O)c(O)c2)oc2cc(O)cc(O)c12	quercetin
CNP0298404.0	Cc1coc2c1C(=O)C(=O)c1c-2ccc2c(C)cccc12	Tanshinone I
CNP0290260.0	COc1cc(O)c2c(=O)cc(-c3ccccc3)oc2c1	Tectochrysin
CNP0135128.0	Cc1ccc(C(C)C)c(O)c1	THYMOL
CNP0271812.0	O=C1c2ccccc2-n2c1nc1ccccc1c2=O	Tryptanthrin
CNP0168167.0	O=c1oc2cc(O)ccc2c2ccc(O)cc12	urolithin A
CNP0270199.0	COc1cc(C=O)ccc1O	vanillin
CNP0224971.0	COc1c2ccoc2cc2oc(C)cc(=O)c12	Visnagin
CNP0330793.0	COc1c(O)cc(O)c2c(=O)cc(-c3ccccc3)oc12	Wogonin
CNP0215909.0	O=c1c2ccccc2oc2ccccc12	XANTHONE
CNP0073791.0	CC1=C(O)c2ccccc2C(=O)C1=O	Phthiocol
CNP0080018.0	COc1ccc(-c2cc(=O)c3ccc4ccccc4c3o2)cc1OC	DiMNF
CNP0083510.0	O=c1cc[nH]c2c(O)cccc12	Quinoline-4,8-diol
CNP0120779.0	O=C1c2ccccc2C(=O)c2cc3ccccc3cc21	5,12-Naphthacenequinone

CNP0123546.0	O=Cc1c[nH]c2ccccc12	INDOLE-3-CARBOXALDEHYDE
CNP0133028.0	Nc1c(C(=O)O)c2nc3c(C(=O)O)cccc3oc-2cc1=O	Cinnabarinic acid
CNP0153549.0	COc1c2ccoc2c(OC)c2oc(=O)ccc12	Isopimpinellin
CNP0166516.0	COc1ccc(-c2cc(=O)c3cc(OC)ccc3o2)c(OC)c1	6,2',4'-trimethoxyflavone
CNP0169802.0	Oc1c(Cl)c(Cl)c(Cl)c(Cl)c1Cl	pentachlorophenol
CNP0184739.0	COc1cc2ccc(=O)oc2c(O)c1OCC=C(C)C	CAPENSIN
CNP0205465.0	Cc1nc(N)cc2[nH]c3ccccc3c12	Trp-P-2
CNP0206536.0	O=c1c2c(O)cc(O)cc2oc2oc3cc(O)ccc3c12	Lupinalbin A
CNP0211069.0	COc1c2ccoc2cc2oc(=O)ccc12	Bergapten
CNP0230289.0	c1ccc2c(c1)[nH]c1ccccc12	CARBAZOLE
CNP0231418.0	OCCc1c[nH]c2ccccc12	Tryptophol
CNP0279987.0	O=c1c(O)c(-c2ccccc2)oc2ccccc12	3-Hydroxyflavone
CNP0281508.0	c1ccc2occc2c1	BENZOFURAN
CNP0283583.0	c1ccc2c(c1)Cc1c-2ccc2ccccc12	1,2-Benzofluorene
CNP0287703.0	O=c1c(-c2ccc(O)cc2O)coc2cc(O)cc(O)c12	2'-Hydroxygenistein
CNP0290896.0	O=C(O)c1cc(O)c2ccccc2c1O	1,4-Dihydroxy-2-naphthoic acid
CNP0302215.0	Oc1cccc2ccccc12	1-NAPHTHOL
CNP0303808.0	O=C1C=C(O)c2ccccc2C1=O	Lawsone
CNP0306924.0	O=c1c(-c2ccc(O)cc2)coc2cc3c(c(O)c12)OCO3	Irilone
CNP0308395.0	O=S(=O)(O)Oc1c[nH]c2ccccc12	Indoxyl sulfate
CNP0309152.0	O=C1c2ccccc2C(=O)c2c1ccc(O)c2O	Alizarin
CNP0310056.0	c1ccc2c(c1)=c1ccc3c4c(ccc=2c14)=c1ccccc1=3	Indeno[1,2,3-cd]fluoranthene

CNP0320773.0	Oc1ccc2ccccc2c1	2-NAPHTHOL
CNP0328371.0	O=c1oc2cc(O)ccc2c2oc3cc(O)ccc3c12	COUMESTROL
CNP0329876.0	NCCc1c[nH]c2ccccc12	tryptamine
CNP0333328.0	O=C(O)C(=O)Cc1c[nH]c2ccccc12	Indole-3-pyruvic acid
CNP0349190.0	O=C1C(c2c(O)[nH]c3ccccc23)=Nc2ccccc21	Indirubin
CNP0371620.0	Cc1c(N)nc(C)c2c1[nH]c1ccccc12	Trp-P-1
CNP0388833.0	COc1c2occc2c(OC)c2c(=O)cc(C)oc12	khellin
CNP0490967.0	COc1cccc2c1cc([N+](=O)[O-])c1c(C(=O)O)cc3c(c12)OCO3	Aristolochic acid
CNP0564599.0	O=C(O)c1cc(=O)c2cccc(O)c2[nH]1	xanthurenic acid
CNP0600507.0	Nc1nccc(-c2nccc3c2[nH]c2ccccc23)n1	Annomontine
CNP0214931.0	COc1c2occc2cc2ccc(=O)oc12	methoxsalen
CNP0231631.3	C/C=C/c1cc(OC)c2c(c1)[C@@H](C)[C@H](c1ccc3c(c1)OCO3)O2	LICARIN B
CNP0310241.0	COC1=CC=CC(CCC2=CC(O)=CC(OC)=C2)=C1	3'-O-Methylbatatasin III
