# Supplementary material for: AHR/NRF2 Dual Agonist Prediction and Natural Compound Screening Based on Machine Learning: A New Strategy for the Treatment of Atopic Dermatitis
Source: Int J Mol Sci. 2026 Apr 15;27(8):3530. doi: 10.3390/ijms27083530 (PMC13115769; doi:10.3390/ijms27083530)
Supplement: Supplementary file 1 [file ijms-27-03530-s001.zip › supplementary materials.pdf]

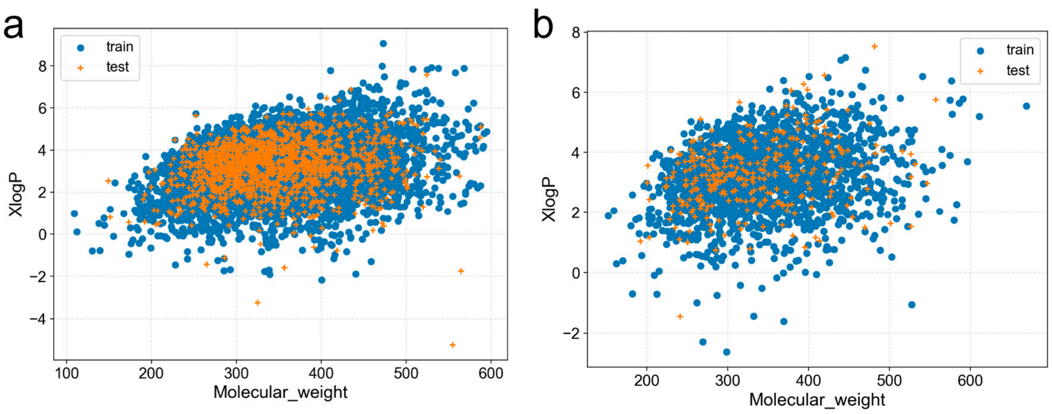

Fig. S1 Scatter plots of compounds' logP and molecular weight. (a) AHR dataset; (b) NRF2 dataset.

Table S1 List of machine learning models used for agonist prediction (AHR and NRF2)

Target	Algorithm	Feature	Name
AHR	RF	E2048	AHR_RF_E2048
AHR	RF	E1024	AHR_RF_E1024
AHR	RF	PUB	AHR_RF_PUB
AHR	RF	MACCS	AHR_RF_MACCS
AHR	LGBM	E2048	AHR_LGBM_E2048
AHR	LGBM	E1024	AHR_LGBM_E1024
AHR	LGBM	PUB	AHR_LGBM_PUB
AHR	LGBM	MACCS	AHR_LGBM_MACCS
AHR	GAT	Graph	AHR_GAT_Graph
AHR	GCN	Graph	AHR_GCN_Graph
AHR	MPNN	Graph	AHR_MPNN_Graph
AHR	AFP	Graph	AHR_AFP_Graph
NRF2	RF	E2048	NRF2_RF_E2048
NRF2	RF	E1024	NRF2_RF_E1024
NRF2	RF	PUB	NRF2_RF_PUB
NRF2	RF	MACCS	NRF2_RF_MACCS
NRF2	LGBM	E2048	NRF2_LGBM_E2048
NRF2	LGBM	E1024	NRF2_LGBM_E1024
NRF2	LGBM	PUB	NRF2_LGBM_PUB
NRF2	LGBM	MACCS	NRF2_LGBM_MACCS
NRF2	GAT	Graph	NRF2_GAT_Graph
NRF2	GCN	Graph	NRF2_GCN_Graph
NRF2	MPNN	Graph	NRF2_MPNN_Graph
NRF2	AFP	Graph	NRF2_AFP_Graph

Abbreviations: E2048, ECFP4-2048; E1024, ECFP4-1024; PUB, PubChem fingerprint; MACCS, MACCS fingerprint; Graph, featurizer of general graph convolution networks for molecules.

Table S2 Hyperparameters of different ensemble models

		AHR				NRF2			
Model	Hyperparameters	E2048	E1024	MACCS	PUB	E2048	E1024	MACCS	PUB
RF	n_estimators	188	126	129	109	160	100	108	100
	max_depth	19	20	19	14	19	-1	19	-1
LGBM	max_depth	6	6	6	7	5	5	7	6
	lambda_l1	0.397	2.218	0.831	0.398	1.272	1.199	0.868	0.527
	learning_rate	0.081	0.09	0.088	0.084	0.093	0.088	0.099	0.093
	min_gain_to_split	0	0	0.004	0	0	0	0	0

Table S3 Hyperparameters of different neuro-network models

	Model	batch_size	learning_rate	dropout	graph_feat_size
AHR	GCN	32	0.001	0.2	--
	GAT	128	0.005	0.2	--
	MPNN	64	0.001	0.5	--
	AFP	64	0.0005	0.5	128
NRF2	GCN	64	0.001	0.2	--
	GAT	64	0.01	0.2	--
	MPNN	32	0.001	0.5	--
	AFP	128	0.001	0.5	--

Table S4 Results from 5-fold cross-validation on the training set

Models	ACC	PR	RE	F1	AUC
AHR_RF_E2048	0.803	0.786	0.834	0.809	0.877
AHR_RF_E1024	0.805	0.791	0.83	0.81	0.88
AHR_RF_PUB	0.791	0.78	0.811	0.795	0.87
AHR_RF_MACCS	0.793	0.771	0.834	0.801	0.875
AHR_LGBM_E2048	0.811	0.801	0.828	0.814	0.883
AHR_LGBM_E1024	0.808	0.796	0.829	0.812	0.882
AHR_LGBM_PUB	0.794	0.781	0.819	0.799	0.866
AHR_LGBM_MACCS	0.809	0.798	0.828	0.813	0.882
AHR_GAT_Graph	0.816	0.875	0.737	0.8	0.915
AHR_GCN_Graph	0.784	0.746	0.86	0.799	0.869
AHR_MPNN_Graph	0.872	0.88	0.862	0.871	0.944
AHR_AFP_Graph	0.848	0.808	0.913	0.857	0.929
NRF2_RF_E2048	0.826	0.822	0.832	0.827	0.899
NRF2_RF_E1024	0.832	0.833	0.831	0.832	0.905
NRF2_RF_PUB	0.805	0.807	0.803	0.805	0.88
NRF2_RF_MACCS	0.823	0.815	0.838	0.826	0.9
NRF2_LGBM_E2048	0.839	0.83	0.851	0.841	0.908
NRF2_LGBM_E1024	0.839	0.83	0.854	0.842	0.905

NRF2_LGBM_PUB	0.808	0.8	0.822	0.811	0.885
NRF2_LGBM_MACCS	0.845	0.837	0.96	0.848	0.91
NRF2_GAT_Graph	0.864	0.92	0.798	0.854	0.954
NRF2_GCN_Graph	0.818	0.879	0.737	0.802	0.899
NRF2_MPNN_Graph	0.935	0.917	0.957	0.937	0.981
NRF2_AFP_Graph	0.912	0.927	0.894	0.91	0.976
